# What matters for lac repressor search *in vivo*—sliding, hopping, intersegment transfer, crowding on DNA or recognition?

**DOI:** 10.1093/nar/gkv207

**Published:** 2015-03-16

**Authors:** Anel Mahmutovic, Otto G. Berg, Johan Elf

**Affiliations:** Department of Cell and Molecular Biology, Science for Life Laboratory, Uppsala University, 75124 Uppsala, Sweden

## Abstract

We have investigated which aspects of transcription factor DNA interactions are most important to account for the recent *in vivo* search time measurements for the dimeric *lac* repressor. We find the best agreement for a sliding model where non-specific binding to DNA is improbable at first contact and the sliding LacI protein binds at high probability when reaching the specific O_sym_ operator. We also find that the contribution of hopping to the overall search speed is negligible although physically unavoidable. The parameters that give the best fit reveal sliding distances, including hopping, close to what has been proposed in the past, i.e. ∼40 bp, but with an unexpectedly high 1D diffusion constant on non-specific DNA sequences. Including a mechanism of inter-segment transfer between distant DNA segments does not bring down the 1D diffusion to the expected fraction of the *in vitro* value. This suggests a mechanism where transcription factors can slide less hindered *in vivo* than what is given by a simple viscosity scaling argument or that a modification of the model is needed. For example, the estimated diffusion rate constant would be consistent with the expectation if parts of the chromosome, away from the operator site, were inaccessible for searching.

## INTRODUCTION

The DNA sliding model for target search was originally formulated to explain the observed rapid rate of binding of lac-repressor from *Escherichia coli* to its operator site on DNA, which was estimated to be ca 100-fold faster than the expected maximal diffusion-limited value (1–4) *in vitro* at low salt concentrations. In the model, the protein finds its target site on DNA through a combination of 3D diffusion and 1D diffusion (sliding) along the DNA contour. The main effect is that the target size is effectively extended from one base pair to the distance that the protein can slide during a non-specific binding event. The model was successful in explaining the high association rate as well as its dependence on the salt concentration, non-specific binding constant and DNA-chain length (5–8). Since its original formulation, the sliding model has been revisited and extended (9–35) and also complemented with informative simulations at levels of detail ranging from atomistic (36,37) to coarse grained (13,17,21,38–39). This has not only generated new insight into e.g. the role of water molecules, condensed ions and DNA conformation during the sliding process but has also stimulated new hypotheses related to search kinetics such as the role of disordered tails in the search (21,38) or the role of multiple DNA binding domains (13,40).

When confronted with *in vivo* data (41), the basic model, which was developed to describe an *in vitro* situation, may need to be extended with features found in the *in vivo* situation. The search problem involves finding a single (or a few) specific site(s) among a vast excess of non-specific ones. The search efficiency will depend on the speed with which the repressor can transfer from one potential site to another and several different mechanisms have been suggested. There are in principle four different ones, correlated or uncorrelated with or without dissociation from DNA. In the correlated search the repressor will search sites nearby on the contour and in an uncorrelated one it will search between random locations. While the repressor remains non-specifically bound it can slide in a 1D diffusion along the DNA contour and test correlated nearby sites, or it can be transferred directly to an uncorrelated DNA segment (intersegment transfer) (5,19,42,43) that is close by in 3D space. Similarly, after a dissociation event, the repressor can rebind at a correlated site nearby (hopping) (5,8) or it can rebind to a different segment (intersegmental jump) (22,31). In addition, there is the possibility that the repressor does not recognize its specific site once it has been found. A specific recognition step is needed, for example, to explain the ca 20% difference in rate between the natural operator O_1_ and a synthetic operator sequence O_sym_ (41). This recognition step is equivalent to introducing a two-step model where LacI is in equilibrium between a non-specific and a specific binding mode conformation (6,29).

Here, we evaluate how these different factors interplay and, in particular, which of the many parameter combinations are more likely to explain the *in vivo* search time measurements for the *lac* repressor dimer binding its symmetric operator O_sym_. The measurements that we seek to account for are the total search time as well as the reduced association rate per operator when two operators are close or when a single operator is flanked by other proteins at specific distances (41). Specific questions that we address are whether there are any physically reasonable sets of parameters for which the model can explain the observed *in vivo* measurements and whether hopping and intersegmental transfers contribute to the search efficiency in this parameter range.

## MATERIALS AND METHODS

### The model

**Figure 1.**
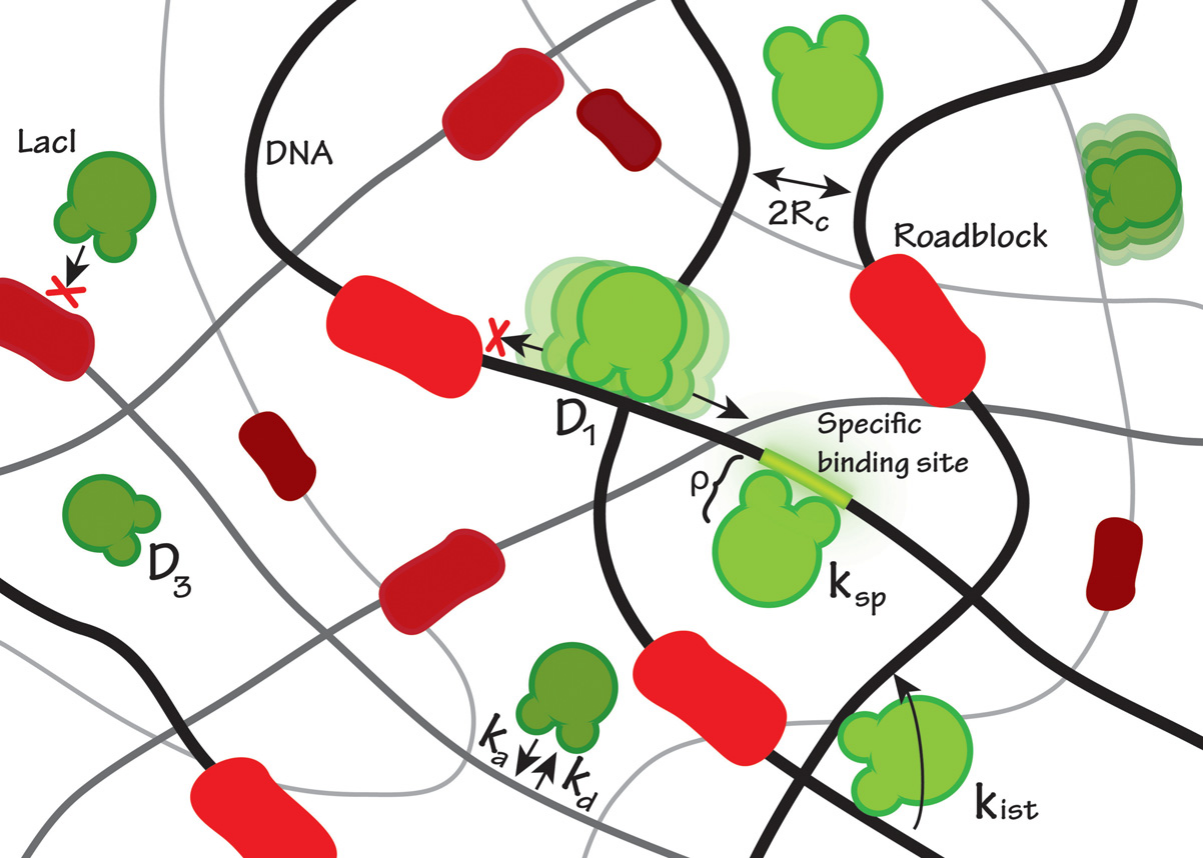
The figure depicts a cartoon of the search process and the fundamental parameters involved in our model. In the MC simulation scheme, following a dissociation the searching protein can associate with rate *k_a_* to a non-specific stretch of DNA given that no other proteins (roadblocks) are in the way. Given a successful non-specific association the protein may diffuse in 1D along the length of the DNA or perform intersegment transfer with rate *k*_IST_. After a number of non-specific association–sliding–dissociation iterations during the total time τ, the searching protein finds its way to the specific binding site and binds with rate constant *k*_sp_.

We build on the classical sliding model (2–5,8) (Figure 1), where the DNA is considered as a smooth cylinder and the protein is a fully reactive sphere. In the Supplementary material the classical model is extended to include roadblocks, i.e. non-specifically bound proteins to DNA, and a specific recognition step at the specific operator site. The reaction radius *ρ* is the sum of the radii of the cylinder and the sphere. Non-specific protein–DNA binding is described by the reaction radius *ρ*, a relative diffusion rate *D*_3_ and the extent of diffusion control, *α* (Equation (3)). All steric effects in the binding are assumed to be incorporated in the parameters *α* or *ρ*. Beyond the distance *R_c_* from the DNA axis, where it is equally far to another DNA segment, the protein is assumed to have lost its correlations with a particular DNA segment and will be equally likely to bind anywhere on the DNA. If the number of accessible non-specific binding sites on DNA is *M*_acc_ with each base pair having a length *ℓ* and where the genome is confined to a volume *V_c_, R_c_* can be determined from

(1) \begin{equation*} M_{{\rm acc}} \ell \pi R_c^2 = V_c. \end{equation*}

This corresponds to a regular distribution where competing DNA segments are considered as lined up in parallel at a mutual distance of ca 2*R_c_*. In the Supplementary material we show that very similar results follow from the assumption that the DNA segments are homogeneously distributed throughout the volume *V_c_*.

A non-specifically bound protein will dissociate with the (microscopic) rate constant *λ*, which leaves the protein in solution at the reaction radius *r* = *ρ*. The motion of the protein after making a microscopic dissociation is described by diffusion in cylindrical coordinates, with a radiation boundary condition at the reaction radius (44) describing a rebinding event,

(2) \begin{equation*} 2\pi D_3 \rho \left. {\frac{{\partial c}}{{\partial r}}} \right|_{r = \rho } = \kappa c(\rho ), \end{equation*}

and an adsorbing condition at *r* = *R_c_*. *κ* is a measure for the reactivity by which the protein will adsorb or bind to DNA at contact.

Solving the diffusion equation for the concentration *c*(*r*) with these boundary conditions the total return probability to the same DNA segment is given by (3–5,8)

(3) \begin{equation*} \phi_0 = \frac{{\alpha \ln \left( {R_c /\rho } \right)}}{{1 + \alpha \ln \left( {R_c /\rho } \right)}}. \end{equation*}

The parameter *α* serves as a measure for the extent of diffusion control of the binding reaction and is defined as

(4) \begin{equation*} \alpha = \frac{\kappa }{{2\pi D_3 }} = \frac{k}{{2\pi D_3 \ell }}. \end{equation*}

Here *k* = κ *ℓ* has been introduced such that *k* is a proper bimolecular association rate constant per base pair; it is the macroscopic rate constant the protein would have if it was diffusing infinitely fast. For *α* >> 1, the non-specific association is significantly limited by the diffusion rate, i.e. it binds non-specifically at the first contact.

In Equation (3), the fraction 1−*ϕ_0_* of microscopic dissociations goes beyond *r* > *R_c_* and will eventually rebind at a random position on the DNA. Thus the macroscopic non-specific dissociation rate can be defined as

(5) \begin{equation*} k_d = \lambda \left( {1 - \phi_0 } \right) = \frac{\lambda }{{1 + \alpha \ln \left( {R_c /\rho } \right)}}. \end{equation*}

To give the same equilibrium constant as the microscopic rates, the macroscopic association rate constant, *k_a_*, must satisfy *k_a_*/*k_d_* = *k*/$\lambda$. Thus,

(6) \begin{equation*} k_a = k\left( {1 - \phi_0 } \right) = \frac{k}{{1 + \alpha \ln \left( {R_c /\rho } \right)}} = \frac{{2\pi D_3 \ell }}{{1/\alpha + \ln \left( {R_c /\rho } \right)}}. \end{equation*}

This is the average association rate constant for a protein starting anywhere in the solution.

At equilibrium the fraction *F_B_* of proteins that will be bound is determined by the non-specific binding constant *K*_RD_ such that

(7) \begin{equation*} K_{{\rm RD}} = \frac{k}{\lambda } = \frac{{k_a }}{{k_d }} = \frac{{F_B }}{{1 - F_B }}\frac{{V_c }}{{M_{{\rm acc}} }}. \end{equation*}

During the time 1/*k_d_* that the protein remains non-specifically bound macroscopically, it will make $\lambda$/*k_d_* −1 = $\phi_0$/(1−$\phi_0$) returning micro-dissociations. For each of these it can diffuse and rebind at a position *z* some distance away, as given by the probability density *F*(*z*) in ((4,5); Supplementary Equation (3)). This process, which is an integral part of the diffusion geometry, has been called hopping.

While non-specifically bound, the protein can move along DNA in a 1D diffusion with rate *D*_1_ (bp^2^s^−1^). If we, for a moment, neglect the displacements to near-by sites during the short-lived hopping events, the protein remains non-specifically bound during the macroscopic residence time 1/*k_d_*. As a consequence, the protein will scan a distance along the DNA corresponding to the sliding length during each macroscopic non-specific binding event

(8) \begin{equation*} s = \sqrt {D_1 /k_d }. \end{equation*}

Thus a protein landing within a distance *s* from the specific site will also find it with high probability, giving an effective target size of 2*s* bp. Actually, under these conditions one finds the total search time (Supplementary Equation (29))

(9) \begin{equation*} \tau = \frac{{M_{{\rm acc}} }}{{2F_B \sqrt {D_1 k_d } }}. \end{equation*}

This is the time to locate the specific site. However, it is possible that the protein does not recognize it and binds but instead slides off. If the protein binds specifically with a finite rate constant *k*_sp_, the total time to bind will be given by the search time (Equation (9)) increased by the factor (41) (Supplementary Equation (31))

(10) \begin{equation*} f_{{\rm sp}} = \left( {1 + \frac{{2\sqrt {D_1 k_d } }}{{k_{{\rm sp}} }}} \right). \end{equation*}

Another complication is that sliding could be impeded due to crowding by other proteins that are bound to the DNA. On the assumption that a fraction *v* (vacancy) of the non-specific DNA is free of bound proteins, Li *et al*. (45) showed that the fraction of sites available for binding by a particular protein is $ve^{1 - \frac{1}{v}}$. Thus, the total number of accessible non-specific binding sites in the genome would be

(11) \begin{equation*} M_{{\rm acc}} = M_{{\rm tot}} ve^{1 - \frac{1}{v}}, \end{equation*}

where *M*_tot_ (bp) is the total genome size. Furthermore, crowding impedes the sliding and the effective target size becomes (45) *L*(*v*) as given by Supplementary Equation (40) rather than 2*s*. Thus the search time in Equation (9) is increased due to crowding by the factor

(12) \begin{equation*} f_{{\rm cr}} = \frac{{2s}}{{L(v)}} = e^{1/v - 1} \sqrt {1 + \frac{{1 - v}}{{vd}}\sqrt {\pi \frac{{D_1 }}{{k_d }}} }. \end{equation*}

Here, *d* is the footprint of the DNA-binding protein. In the parameter regime where the recognition step and the effects of crowding contribute independently to the search time, both correction factors (Equations (9) and (12)) can simply be multiplied to the result (Equation (9)). In this case the search time is given by

(13) \begin{eqnarray*} &&\tau = \nonumber \\ &&\frac{{M_{{\rm tot}} }}{{2F_B }}\sqrt {\frac{1}{{D_1 k_d }}\left[ {1 + \frac{{1 - v}}{{vd}}\sqrt {\pi \frac{{D_1 }}{{k_d }}} } \right]} \left( {1 + \frac{{2\sqrt {D_1 k_d } }}{{k_{{\rm sp}} }}} \right). \end{eqnarray*}

This simple approximation works well in the parameter space investigated as shown both by the simulations (Supplementary Figure S7) and by theory (Supplementary Equation (44)). However, with two operator sites within sliding distance of each other, it is more difficult to find analytical solutions for the combined effects of crowding on DNA, hopping and finite binding rates and we have therefore resorted to computer simulations to test the corresponding parameter combinations. The simulations recover the analytical results in the appropriate limits as shown in Supplementary Figure S7.

The effect of intersegment transfer is to modify the exchange rate between DNA segments following a macroscopic dissociation, *k_d_*, by adding to it another exchange rate *k*_IST_ (5). The nature of *k*_IST_ is to allow for an exchange between DNA segments without prior dissociation. We therefore make the substitution *k_d_ → k_d_*+*k*_IST_ in Equations (8) - (13) without changing the fraction bound *F_B_*.

### The Monte Carlo simulation scheme

The search process for one or two operator sites was realized using a 1D 5000-element (base pairs) lattice MC simulation with periodic boundary conditions representing the DNA. A protein corresponds to a position on the lattice with all proteins having a footprint *d*, where *d* was set to 21 bp. Except for the searching protein, the other proteins involved are nucleoid associating (NAPs) such as H-NS, Fis or IHF in *E. coli* (46). It is assumed that the NAPs are stationary during the time microscopic processes (hopping and sliding) govern the target search on the ms time scale. It has previously been shown that the effect of allowing NAPs to move during the microscopic sliding events is small (45). When the searching protein dissociates macroscopically the NAPs are uniformly redistributed on the lattice. The redistribution frequency is justified by the fact that the differences between the dissociation constants for LacI and NAPs under comparable *in vitro* conditions (6,42,47–49) are mostly within an order of magnitude of each other. We tested this by redistributing NAPs every 10th LacI macroscopic dissociation which resulted in a difference in search times within a few percent depending on the vacancy. The output of the simulation is the average number of microscopic or macroscopic binding–dissociation cycles before one of the two operators is found in the 5000-bp DNA. The number of cycles is then linearly scaled to the 4.5 × 10^6^-bp *E. coli* chromosome.

The algorithm starts with randomly positioning the searching protein on DNA followed by placements of roadblocks, one on each side of LacI such that the end-to-end distance (*ζ*) distribution between them is *p*(*ζ*) (45) (Supplementary Equation (45)). A new end-to-end distance distribution is created by multiplying *p*(*ζ*) with (*ζ*−*d*+1) where *ζ =* {*d,d*+1…,2500ζ−*d*} since the simulations condition on LacI being placed on DNA before the roadblocks. There is exactly one way to place LacI between the roadblocks if the distance between them is *d*, two ways if the distance is *d*+1 and so on. The contribution to the search time from the binding attempts hindered by obstructing proteins is taken care of in the dissociation rate in Equation (16) through the definition of the equilibrium constant (Equation (7)), which contains the number of accessible sites only. This approach where two roadblocks are distributed at either side of the protein with the end-to-end distance distribution as given in (45) agrees very well with simulations where all roadblocks are included.

Next step in the simulation is to sample whether the searching protein should (i) dissociate microscopically with rate λ, (ii) slide 1 bp in a direction which is not blocked by another protein with rate *D*_1_, (iii) bind specifically with rate *k*_sp_ if the protein is on the lattice corresponding to the specific site or (iv) intersegment transfer with rate *k*_IST_. The probability for each event corresponds to the fractional rate of the event as for any time continuous Markov process; e.g. when the protein can slide in two directions the probability that the next event is a microscopic dissociation is

(14) \begin{equation*} p_{{\rm dissoc}} = \frac{\lambda }{{\lambda + 2D_1 + k_{{\rm sp}} + k_{{\rm IST}} }}, \end{equation*}

or a specific binding event

(15) \begin{equation*} p_{{\rm bind}} = \frac{{k_{{\rm sp}} }}{{\lambda + 2D_1 + k_{{\rm sp}} + k_{{\rm IST}} }}. \end{equation*}

Using the experimentally determined values for *M*_tot_, *V_c_, D*_3_, *ρ, F_B_, v* and *ℓ* as constants and the largely unknown *D*_1_, *α* and *p*_bind_ as variable input parameters, *λ* and *k*_sp_ can be solved for and *p*_dissoc_ calculated; see Table 1 for a summary of the parameter names and their definitions in the model.

**Table 1.** Description of the parameters used in the model

*M*_tot_ (4.5 Mbp)	Total *E. coli* genome size
*V_c_ (*1 μm^3^)	Cell volume per genome equivalent
*D*_3_ (3 μm^2^s^−1^)	3D diffusion coefficient for LacI
*ρ* (5.5 nm)	The reaction radius between LacI and a non-specific DNA segment
*ℓ* (0.34 nm)	The width of one base pair
*F_B_* (0.9)	The fraction of time a searching protein stays non-specifically bound to DNA
*v* {0.70,0.85}	The fraction of the genome unoccupied by DNA binding proteins
*D*_1_ {0.01–0.05} μm^2^ s^−1^	1D diffusion coefficient for a non-specifically bound LacI molecule
*α* {0.05–0.80}	The degree of diffusion control
*p*_bind_ {0.2,1.0}	Probability of binding the specific operator site
*k*_IST_ {0–500}s^−1^	Intersegment transfer rate

The variable parameters are the 1D diffusion coefficient *D*_1_ [μm^2^s^−1^], the degree of diffusion control, *α*, the probability of binding the specific site given that the searching protein is on it, *p*_bind_, are indicated by the curly brackets. The other parameters are considered well known and are fixed except for the vacancy on DNA, *v*, where we test two different values.

Using Equations (14) and (15) we determine whether to dissociate microscopically or bind specifically. Given a microscopic dissociation the searching protein has a chance of macroscopically dissociating as given by Equation (3). A macroscopic dissociation implies resampling the position of the searching protein and all the crowding proteins on the DNA.

If the protein dissociates microscopically, but not macroscopically, and hopping is included in the description, then the protein is displaced along the length of the DNA according to the distribution in Supplementary Equation (3) with reflective boundary conditions at the edge of a roadblock; if hopping is not included, the protein is simply returned to the site it came from. The number of microscopic dissociation events until specific binding is recorded, and the whole process is repeated 150 000 times to get a good estimate of the average search time for one set of parameters.

To arrive at the total search time, the number of microscopic association–dissociation cycles (*N*_micro_) is multiplied by the time it takes for each cycle, i.e. $\left( {\lambda F_B } \right)^{ - 1}$. Thus,

(16) \begin{equation*} \tau = \frac{{N_{{\rm micro}} }}{{\lambda F_B }} = \frac{{N_{{\rm macro}} }}{{(1 - \phi_0 )\lambda F_B }}. \end{equation*}

## RESULTS

### Approach

**Figure 2.**
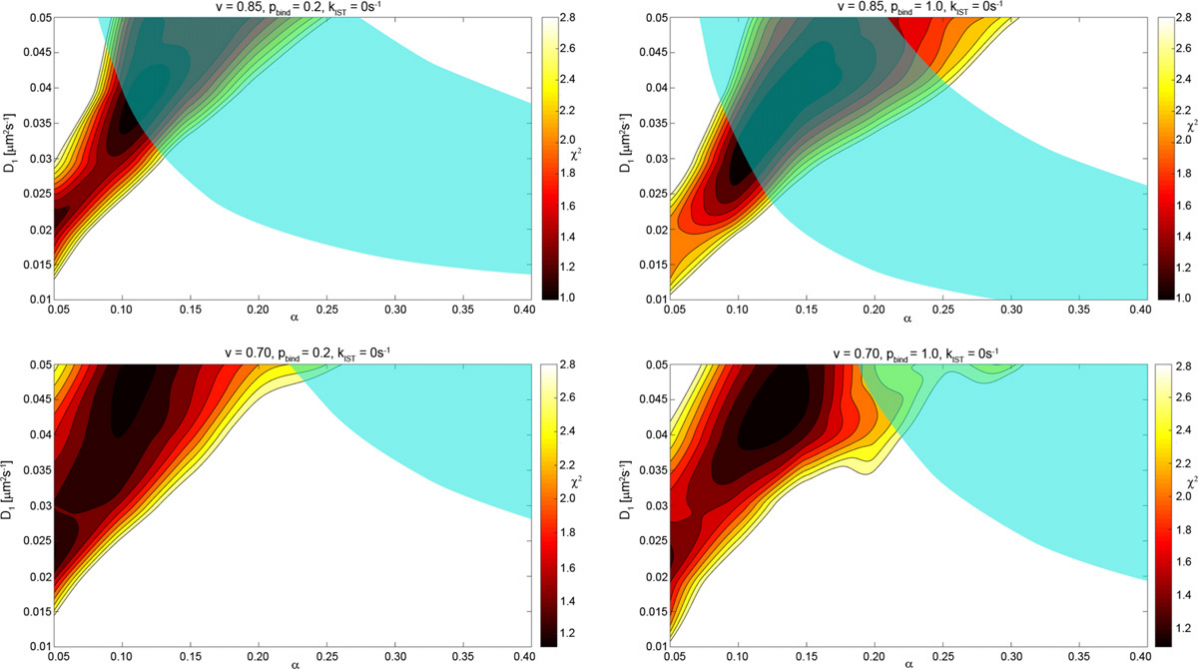
The level curves correspond to chi-square values less than 3 measuring the goodness of fit of simulations to experiment for one of the two operator sites at distances 25, 45, 65, 115 and 203 bp. The values are calculated for combinations of *D*_1_ and *α* values given the vacancy *v*, the probability of specific binding *p*_bind_ as indicated and the fraction of non-specific binding *F_B_* equal to 90% (Table 1). The cyan region in this figure shows where the absolute search time is in the interval 236 s (three proteins)–416 s (five proteins).

**Figure 3.**
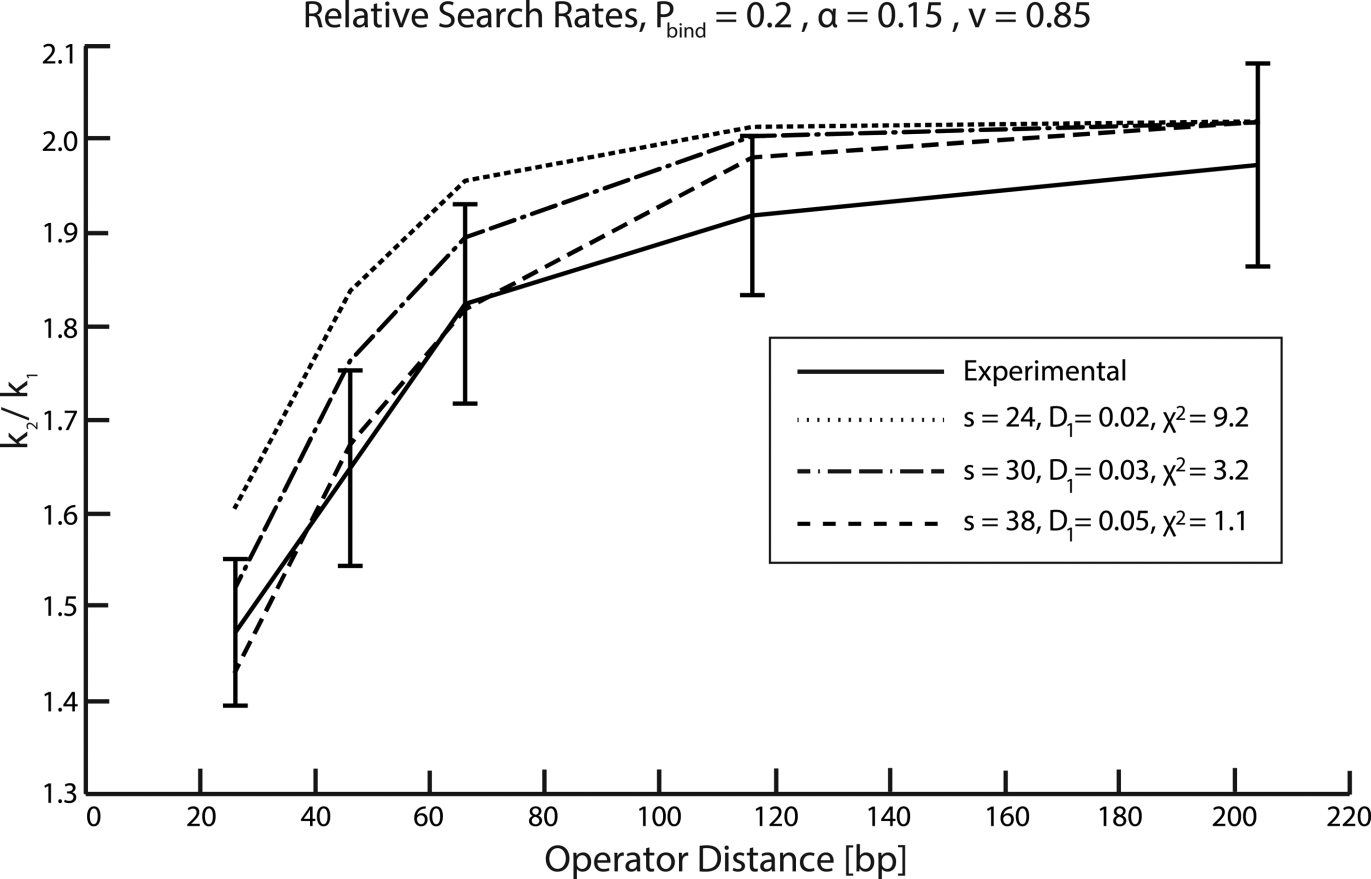
The figure shows examples of fits of simulations (dashed, dash-dot and dotted curve) to experiments (solid curve) and the corresponding chi-square values used as constraints in determining the solution space. The y-axis is the ratio between the rate with which LacI finds one of the two operator sites at a certain distance from each other and the rate with which it finds one operator site; the x-axis is the distance between operator sites in base pairs. The *s*-values in the legend are the unobstructed sliding length for LacI given by Equation (8).

Our approach to estimate the *in vivo* parameters governing the target search is to acquire the time it takes for LacI to find its specific binding site using a combination of Monte Carlo simulations and analytical calculations as a function of three unknown variable input parameters *D*_1_, *α* and *p*_bind_. The calculated search time is then used to compare with two sets of single molecule *in vivo* data which are the search times to one operator site and the ratio of search times for one and two operator sites at various distances. The parameter regions where there is agreement between the calculated search times and the measured search times are illustrated in Figure 2. Here the semi-transparent cyan region corresponds to parameters where the overall search time to one site is acceptable and the level curves correspond to chi-square values for the agreement for the two-operator data set. The calculated search times should conform to both sets of *in vivo* data, which means that the acceptable parameter space is the region where the cyan region overlaps with the contour map. The constraint to small chi-squared values is because they quantify the difference between the estimated search times and the experimental search times for the data involving two operator sites as exemplified in Figure 3. This approach is expanded upon and clarified in the sections that follow.

#### Parameters

We treat the parameters that are not known *in vivo* as variable input parameters (see Table 1), i.e. the probability (*p*_bind_) that the repressor recognizes and binds to its specific site after finding it, the 1D diffusion coefficient for sliding along DNA *in vivo* (*D*_1_ given in bp^2^s^−1^ in the equations, otherwise given in μm^2^s^−1^) and the degree of diffusion control in binding non-specific DNA sequences (*α*). The parameters, for which we have better estimates, are treated as constants, i.e. the 3D diffusion coefficient for LacI *D*_3_ = 3 μm^2^s^−1^ (50), the *E. coli* genome size set to *M*_tot_ = 4.5 × 10^6^ bp, the base pair length *ℓ* = 0.34 nm, the cell volume (per genome equivalent) *V_c_* = 1 μm^3^, the reaction radius *ρ* = 5.5·10^−3^ μm and the fraction of time the searching protein stays non-specifically bound *F_B_* = 0.9 (41). For the fraction of the total DNA length not covered by other proteins (i.e. the DNA vacancy *v*) there are alternative literature values and we therefore independently tested both *v* = 0.7 (45) and *v* = 0.85 (46). Furthermore, the average number of repressors per genome equivalent in the experimental system was estimated (41) to be between 3 and 5.

Two parameters, *α* and *D*_1_, were varied systematically given high and low values of *p*_bind_. *p*_bind_ is given the extreme value *p*_bind_ = 1, corresponding to always binding when reaching the site and *p*_bind_ = 0.2, below which we get no solutions with an acceptable total search time. The range of *D*_1_ is confined from above by the corresponding *in vitro* value of 0.046 μm^2^s^−1^ (50,51) and from below by 0.01 μm^2^s^−1^ where the total association is too slow to give any acceptable solutions. Based on viscosity effects alone, Tabaka *et al*. (52) calculate *D*_1_ = 0.025 μm^2^s^−1^; this neglects friction with DNA and corresponds to half the *in vitro* value. Thus, we expect the actual value to be lower than this. *α* can, in principle, take any real positive value and we evaluate the model in the range where we have acceptable agreement with both sets of data.

#### Correlated binding to two different operator sites

The most informative experimental constraints on the allowed parameter space are those that compare the ratios of the search times for one of the two specific sites at distances 25, 45, 65, 115 and 203 base pairs apart and one specific site alone (41). When the specific sites are far apart, the approaching protein will see them as two independent sites, but when they are within sliding distances from each other, the effective target will look more like a single site. The level curves correspond to chi-squared values less than 3. Figure 3 shows examples of the fits afforded for some of these chi-square values justifying our choice of the threshold at 3 as a good-fit region.

The positive correlation for low chi-square values (red) in the *D*_1_-*α* plane of Figure 2 is due to the opposite effects these parameters have on the sliding length (Equation (8)). Increasing the degree of diffusion control means increasing both association and dissociation rates (Equation (4)) since the equilibrium constant is held constant. The sliding length, being inversely proportional to the square root of the dissociation rate, will thus decrease, which means that *D*_1_ increases to maintain the sliding length and the dependence between two operators at different distances.

#### The total binding time

In the Materials and Methods section we derive the equation for the total search time for one repressor and one operator when crowding and finite binding probability at the operator site is introduced (Equation (13)). As the derivation includes some approximations, the validity of this equation was tested and found to agree well with simulations in the parameter ranges of interest (Supplementary Figure S7).

The total search time from this analytical expression is used as an additional constraint for the acceptable parameter space. The average number of searching proteins was estimated to be between 3 and 5 per genome equivalent (41) with an experimental search time for O_sym_ of 81 ± 2 s. This corresponds to a search time for a single protein to fall between 236 and 416 s. In Figure 2 this is translated to an acceptable parameter space with respect to the absolute search time shown in cyan. An agreement between experiments and the model would be achieved for parameter values where the cyan region overlaps with the ‘good-fit’ red chi-square values. From the figure it is evident that the model is not consistent with the calculated value *v* = 0.7, since it would require *D*_1_ > 0.04 μm^2^s^−1^ for a reasonable fit. This lends more credibility to the alternatively reported value 0.85.

### Constraints from one or two stationary roadblocks

Another experimental result that can be used to constrain the solution space is the ratio of the search times with and without a stationary protein, a roadblock, positioned next to the operator site (see Table 2). The roadblock should increase the binding time by a factor of two if the DNA is naked except for the roadblock and *p*_bind_ = 1.The ratio between the times has been found to be less than 2 *in vivo*, which was one of the arguments suggesting that *p*_bind_ < 1 (41). In the presence of other semi-dynamic roadblocks however, this argument fails. A stationary roadblock next to the operator reduces the probability that a random roadblock will overlap the operator and thereby hinder a specific binding event. The increased influx to the operator site counteracts the doubling of the search time one would expect when only considering that half of the pathways into the operator site is cut off by the stationary roadblock. This can explain a factor less than 2 also when *p*_bind_ equals 1. Further, the values in Table 2 show that the magnitude of the influx effect increases (the ratios get lower) as the vacancy decreases. This is due to a relative increase in the number of events where roadblocks are excluded from the specific operator site. When *p*_bind_ is 1, the increase in search time is simply a factor 2*v* (Supplementary Equation (47)) and the results (Table 2) exclude the case with 70% vacancies in the operator region.

**Table 2.** Stationary roadblock next to the operator site, simulations and analytic results

	Simulations	Theory
(*p*_bind_ = 1.0*, v* = 0.85)	1.66	1.70
(*p*_bind_ = 0.2*, v* = 0.85)	1.60–1.50	1.63–1.55
(*p*_bind_ = 1.0*, v* = 0.70)	1.35	1.40
(*p*_bind_ = 0.2*, v* = 0.70)	1.33–1.26	1.37–1.32

The table shows the ratio between the time for binding one specific operator site with and without one stationary roadblock adjacent to the operator site for different values of *p*_bind_, and *v* with *D*_1_ = 0.03 μm^2^s^−1^ and *α* = 0.10–0.80. The experimental value is 1.75 ± 0.18 (41). Results are from simulations and from theory (Supplementary Equation (46)).

Hammar *et al*. (41) also have results for two cases where the operator is boxed in between two stationary roadblocks with different gap length. The simulation results for these cases are consistent with the parameter estimates above but do not contribute any new constraints on their values.

### The sliding length

An important parameter for characterizing the target search is the sliding length (Equation (8)) for which there are both theoretical and experimental estimates (8,41,52). The best fits to the model result in sliding length between 30 and 40 bp for the acceptable solution space i.e. where cyan regions overlap with chi-squared level curves. These sliding lengths are in accord with recent *in vivo* estimates (41,52).

### The effect of hopping

**Figure 4.**
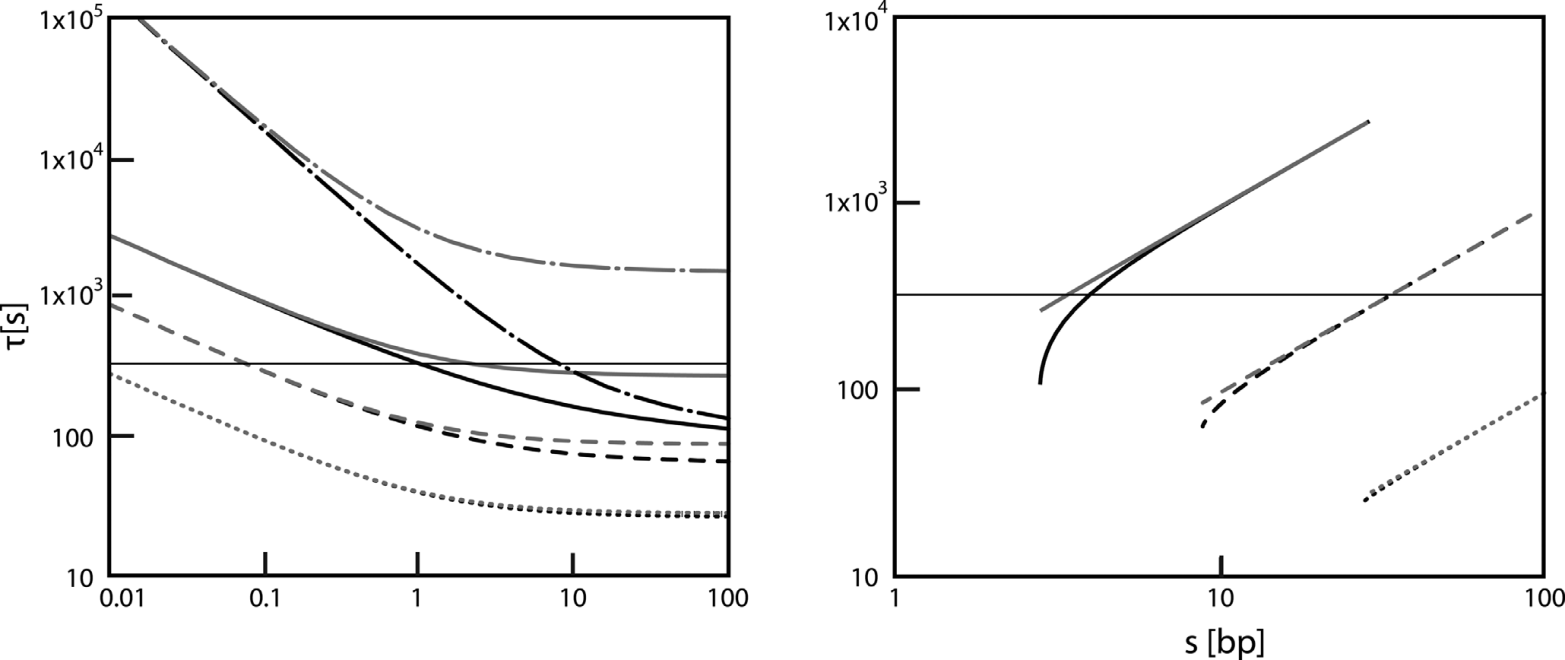
The association time to a specific site *in vivo* calculated for *R_c_* = 0.014 μm, *L* = 765 μm, *D*_3_ = 3 μm^2^/s, *F_B_* = 0.9 and *ρ* = 5.5 nm. Black curves are the full results from Supplementary Equation (27) with hopping and gray curves are approximate neglecting the effects of hopping from Supplementary Equation (29). *D*_1_ = 0.3 (dotted), 0.03 (dashed), 0.003 (solid) and 0 (dash-dot) μm^2^s^−1^. Horizontal gray line shows the measured *τ* of ca. 325 s. Left panel: plotted as a function of α; right panel: as a function of sliding length.

Hopping is the correlated motion of the searching protein along the length of DNA at the microscopic dissociation distance where the protein loses all electrostatic and van der Waals interactions with DNA. The hopping effect is analytically tractable and the theory is developed in the Supplementary material in the case of no crowding proteins (*v* = 1) and unity probability of specific binding (*p*_bind_ = 1). The results are summarized in Figure 4 where we have used the expressions for the total search time with and without hopping in Supplementary Equations (27) and (28), respectively. In the case without hopping a microscopic dissociation results in rebinding to the same position it left. These results only show divergence between the search times with and without hopping when the degree of diffusion control (*α*) is increased beyond 1 or as the sliding length is decreased below 10 for LacI. Thus, we begin to see an increase in the relative contribution of hopping which effectively extends the specific operator site a few base pairs as the sliding length decreases. In the absence of sliding, hopping could decrease the search time by an order of magnitude (if *α* > 10; dashed-dot curves in Figure 4 Left Panel). However, the parameter region where hopping influences the search time falls outside the bounds of the experimental constraints; low *D*_1_ and high *α* means high chi-square values (see Figure 2). Furthermore, the search time remains invariant with respect to hopping also when both crowding proteins and a specific binding probability less than 1 are introduced in the MC simulations (data shown in Supplementary Figure S5) for combinations of parameters that are consistent with experimental data.

### Intersegment transfer

**Figure 5.**
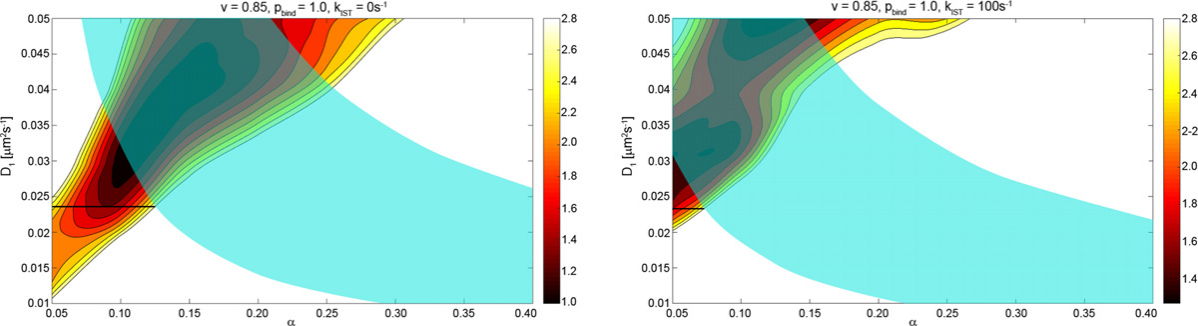
The effect of intersegment transfer on the parameter space for the intersegmental transfer rate, *k*_IST_ = 100. The level curves correspond to chi-square values less than 3 measuring the goodness of fit of simulations to experiment for one of the two operator sites at distances 25, 45, 65, 115 and 203 bp. The chi-squared values come from simulations and the absolute time semi-transparent cyan region was calculated for the best fit combination of parameters with vacancy *v* = 0.85, the probability of specific binding *p*_bind_ = 1.0 and the fraction of non-specific binding *F_B_* is equal to 90%. The cyan region in this figure shows where the absolute search time is in the interval 236 s (three proteins)–416 s (five proteins). The black horizontal lines aid in comparing the lowest attainable *D*_1_ values between the cases of no intersegment transfer (left: *k*_IST_ = 0 s^−1^) and with intersegment transfer (right: *k*_IST_ = 100 s^−1^).

The process by which two DNA segments diffuse within binding distance of a protein capable of transiently binding both segments and therefore transfer from one segment to the other without dissociation is called intersegment transfer. We take this mode into account using the approach of (5). For the combination of parameters having no analytic counterpart we used simulations to get the chi-squared values. We find that intersegment transfer rates below ca 100 s^−1^ have a negligible effect on the minimal acceptable *D*_1_ value (Figure 5). The reason for the small effect is that intersegment transfer disrupts the sliding process which weakens the correlations in the two-operator data effectively increasing the required *D*_1_ value. At the same time, intersegment transfer increases the specific association rate, effectively decreasing *D*_1_. For higher values of *k*_IST_, the fit becomes increasingly bad, pushing the minimal acceptable *D*_1_ higher (Supplementary Figure S8).

## DISCUSSION

In this work we have tested if the classical sliding model for target location, with a few extensions that previously have been discussed and analyzed independently (1D crowding, hopping, finite binding probability and intersegment transfer), is sufficient to explain recent single-molecule *in vivo* data with reasonable parameter values. The free parameters in the simulations are the 1D diffusion coefficient *D*_1_ and the degree of diffusion control *α* which have been varied for high and low *p*_bind_ (the probability of binding specifically at the operator site). The rest of the parameters are taken from experimental literature reports. When there are conflicting literature values we have tested which set of parameters results in the best overall fit. There are also obviously uncertainties for other literature values such as the number of searching proteins *N* or the total accessible genome size *M*_acc_.

Given the limitations, the parameter sweep generates a small but reasonable solution space where the model explains the experimental data, and without additional experimental observations we find no need to discard the model. The solution space does, however, result in relatively specific predictions of the unknown parameters; the most unexpected of these is a relatively high value of *D*_1_ and a low value of *α*.

### Parameter space—1D diffusion and sliding

The upper bound on *D*_1_ was set to 0.05 μm^2^s^−1^ based on *in vitro* single-molecule tracking experiments (50). The parameter sweeps suggest that *D*_1_ cannot decrease below 0.01 μm^2^s^−1^ (Figure 2) to give both the experimental association time and reasonable chi-square values. However, a simultaneous fit with the two-operator dependence suggests that *D*_1_ ≥ 0.02 μm^2^/s. This seems high, considering that viscosity effects alone one gets *D*_1_ close to 0.025 μm^2^s^−1^ *in vivo* (52) which becomes 0.009 μm^2^s^−1^ when multiplied with a retardation factor $e^{ - (\varepsilon /k_B T)^{2 }}$(37) which takes into account that LacI diffuses in a rugged-free energy landscape (53) with roughness ϵ ∼ 1 *k_B_T* (37,53). Thus a *D*_1_ value approaching 0.009 μm^2^s^−1^ is more reasonable than the *D*_1_ ≥ 0.02 μm^2^/s constraint given by the model and the simulations. There are two possible reasons for this apparent discrepancy, either the model or some of its parameters are wrong, which is addressed in the next paragraph, or 1D sliding *in vivo* is faster than what can be expected by an extrapolation from *in vitro* data. This would in particular suggest that the magnitude of the ruggedness of the free-energy landscape (37,53) along DNA could be higher *in vitro* than *in vivo*. A smoother free-energy landscape *in vivo* might be due to a stabilization of the DNA phosphate backbone by nucleoid-associated proteins, metabolites or ions (4,6) missing *in vitro*. Other effects that are neglected in the simple lattice MC simulation method and which could potentially explain the discrepancy between the expected and the fitted *D*_1_ values are hydrodynamic interactions (HIs) and conformational fluctuations (CFs) of DNA (54). These effects enter in the 1D diffusion coefficient (*D*_1_) and the propensity of LacI to rebind (*α*). As these parameters were varied in the simulations and then constrained by fitting to experiments both HIs and CFs of DNA are included to the extent that they contribute to the *in vivo* search kinetics, although we cannot separate each effect and gauge its respective magnitude. However, *D*_1_ calculated from viscosity effects on the protein motion and multiplied with the fluctuations in the interaction free-energy between LacI and DNA gives ∼0.045 μm^2^s^−1^ (51) which is the value measured *in vitro*. Therefore, we expect that the same kind of estimate, without explicitly including the effects of CFs and HIs, will also be valid *in vivo*.

### Parameter space—uncertainties

**Figure 6.**
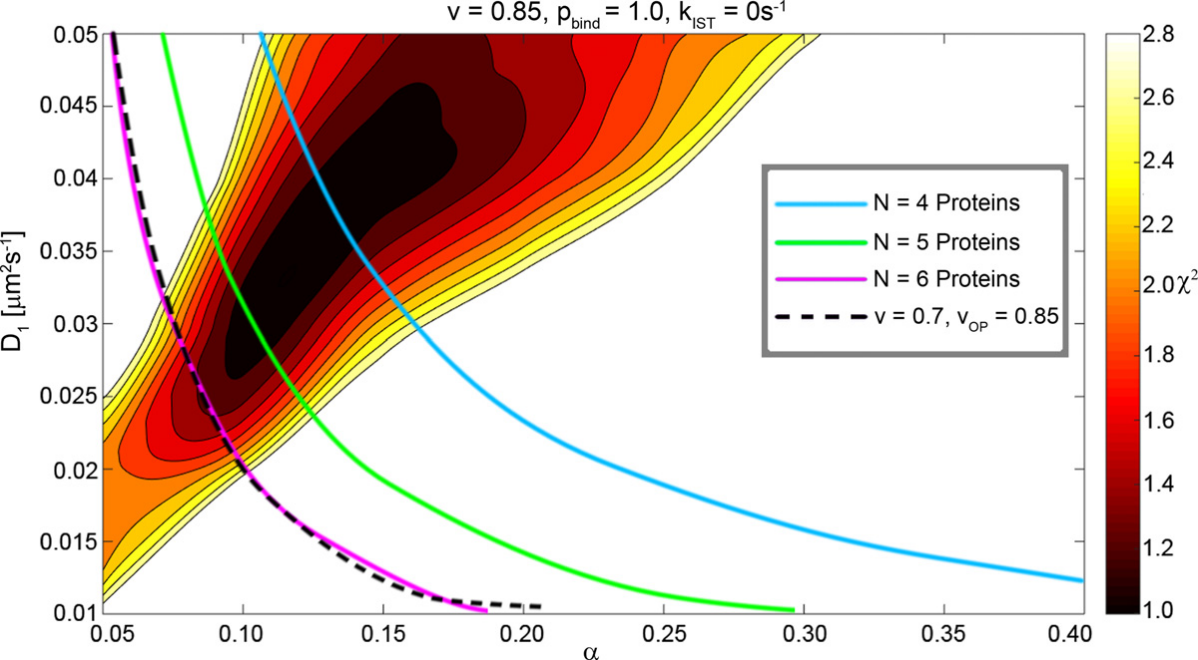
The dependence of the parameter space on the total number of searching proteins and the genome size showing how the front of the semi-transparent cyan regions in Figure 2 would move under different assumptions. The blue, green and magenta lines correspond to 4, 5 and 6 searching proteins, respectively, while the black dashed line represents an 85% vacancy around the operator site (*v*_OP_) and a vacancy of 70% elsewhere. The chi-square values depend weakly on the genome size as tested by simulations. The acceptable 1D diffusion constant is pushed toward lower values by either increasing the number of searching proteins or decreasing the genome size.

When looking at which of the experimental input parameters are most uncertain we find that there are considerable uncertainty in both the DNA vacancy *v* and in the estimated number (*N* ≈ 3–5) of repressors participating in the search. The number of proteins does not influence the chi-square fits in Figure 2, but the total search time is influenced in direct proportion. Thus, if *N* > 5, the overlap region would be pushed toward lower *α* and *D*_1_ (Figure 6). The DNA vacancy has been assumed to be uniform over the chromosome; however, it could be very different around the operator regions as compared to the bulk of the DNA, for instance if parts of the genome are excluded due to local folds or if DNA binding proteins themselves are clustered in regions of the chromosome other than the operator regions (55). This would have the effect of lowering the occupancy around the operator site while allowing for more DNA binding molecules overall. Taking this effect into account, the overlap between the cyan region and the red chi-square values would increase, which would also allow for lower *D*_1_ values (Figure 6).

### Parameter space—crowding and the propensity to bind non-specifically

There are conflicting reports concerning the occupancy of the DNA where Li *et al*. (56) calculate 30% while Tabaka *et al*. (45) calculate 15%. Crowding on DNA has at least three distinct effects on the association process: (i) binding of other proteins on DNA beyond the immediate operator region reduces the amount of competitive DNA leaving less DNA to be searched. Alternatively, if, as in the calculations above, the fraction *F_B_* of non-specifically bound repressor is considered as a given experimental quantity, the reduction of competitive DNA would lead to a larger non-specific binding constant (*K*_RD_). (ii) Crowding molecules can directly obstruct the operator site and thereby reduce the rate of binding. (iii) Finally, crowding molecules would interfere with the sliding process, effectively reducing the distance the repressor can slide along the DNA. Thus when crowding is introduced, the unobstructed sliding distance will have to be increased to explain the dependence in the two-operator data (Figure 2). The solution spaces exclude the previously proposed 30% occupancy case simply because the search becomes too slow. The 30% occupancy cases also conflict with the results from placing a stationary roadblock adjacent to the operator (see Table 2).

As a solution, there could be two levels of chromosomal DNA coverage: for example, perhaps half of the crowding proteins have specific binding sites outside the operator region and would therefore only contribute to the decrease of the competitive DNA. Moreover, this competition could also decrease if some DNAs were inaccessible due to packing in the nucleoid. Thus, the kinetic data suggest that *v* = 0.85 at least in the operator region.

In the calculations above we have used *F_B_* = 0.9, i.e. the repressor spends 90% of its search time on non-specific DNA (41). We have also tried *F_B_* = 0.7 for which we find no acceptable solution space. For proteins with very strong non-specific binding (*F_B_* → 1), intersegment transfer would be needed to avoid having them trapped in DNA regions far from the operator or between roadblocks.

### Parameter space—the recognition step

Once the repressor has reached the operator site it might pass by without recognition and specific binding. Hammar *et al*. (41) introduced an extra recognition step that was given by the probability of binding, *p*_bind_, for a repressor non-specifically bound at the operator site. This parameter was introduced to explain the difference in association rates to operators that differ only a few bases in sequence, in particular the 20% difference in rate between the natural operator O_1_ and a synthetic operator sequence O_sym_ at 298 K; Without this extra step, the sliding model cannot explain why the association rate depends on the operator sequence. A finite recognition step could also explain why blocking off half of the access paths for the repressor by placing a stationary roadblock on one side of the operator does not lead to an increase in the association time by a factor of two but only by 1.75. However, as we have shown in Table 2, such a stationary-roadblock effect also follows from introducing crowding with *v* = 0.85. This is because the stationary roadblock blocks not only the access path for the repressor but also random roadblocks from directly obstructing the operator site. The data used in the present analysis all refer to O_sym_, which consequently could have *p*_bind_ close to 1, while O_1_ could have a significantly smaller value. Indeed, *p*_bind_ = 1 affords a slightly better fit to the data than does *p*_bind_ = 0.2 (Figure 2).

It should be noted that sliding allows the protein to test a site many times before moving away, so that even a small value for *p*_bind_ leads to a modest decrease in the overall association rate (41). For the relative small values of *α* and large values of *s* found here, the effect of *p*_bind_ < 1 is to increase the association time by a factor (Equations (10) and (15)) which can be approximated as $1 + \frac{{1 - p_{{\rm bind}} }}{{p_{{\rm bind}} }}\frac{1}{s}$. This gives roughly a 10% increase for *p*_bind_ = 0.2, but only 2.5% for *p*_bind_ = 0.5. Thus, it would be difficult to distinguish *p*_bind_-values above ∼0.5 from this effect, why we can only say that *p*_bind_ is so high that the overall binding rate to O_sym_ is not significantly reduced.

### Diffusion control and steric effects

The best fit to the *in vivo* kinetic data suggests that the parameter *α* is small (*α* ≈ 0.1–0.15; Figure 2) (52). This would imply that the non-specific association rate constant (*k_a_*) is smaller than its maximal diffusion-limited value, which may be interpreted as a reaction limited non-specific binding. However, the reduction in *k_a_* could also be due to steric constraints, rather than an activation step in the non-specific binding (37). Thus, the non-specific association could well be diffusion limited when the steric effects are accounted for.

The effective reaction radius for non-specific binding, *ρ*, is difficult to estimate. At a maximum *ρ* would correspond to the distance between the mass centers of the DNA cylinder and the protein when they form a non-specific complex (the Smoluchowski limit), i.e. *ρ*_max_ ∼5.5 nm. This would be the case if all molecular collisions would have a high probability of finding the productive orientational configuration required for a bound complex to form before diffusing apart again. This, in turn, would require that the molecules are held together for some time while they explore their mutual orientational space, akin to a local sliding process. Although electrostatic attraction could help pull the reactive regions together into a productive orientation, these forces are screened and will help primarily when the reactive regions are already close. If *α* ∼ 0.1 is interpreted as being due to steric effects (37), then the corresponding effective interaction radius would be *ρ*_eff_ = *ρ*_max_*e*^−1/^*^α^*, which could be on the order of ∼10^−4^ nm as expected from some protein–protein association rates. While the diffusion-limited protein–protein association rate is directly proportional to *ρ*_eff_, for the protein–DNA association, being largely a 2D process, a small value for *ρ*_eff_ decreases the macroscopic association rate only logarithmically.

### Hopping

Hopping is a process where a non-specifically bound protein dissociates and quickly rebinds mostly to the same DNA site but occasionally to one nearby along the DNA contour. Since hopping is a consequence of the diffusive motion in the geometry of the protein leaving DNA in the form of an extended chain, we expect it to exist for all DNA binding proteins. It can be formally distinguished from sliding in that it involves diffusion paths where there are no interactions between DNA and protein. The task at hand is to quantify the influence of hopping on the rate with which the LacI dimer finds its specific binding site. The modeling (5,8) shows that hopping would have little effect on the LacI association process in the presence of sliding (Figure 4). However, hopping would be very important for non-sliding proteins by allowing one that is ‘almost there’ to try neighboring binding sites without having to start the search all over. Although, it would be possible to model the observed specific association time *in vivo* in terms of hopping alone (Figure 4), it cannot explain the observed dependence between different binding sites; since sliding is needed to bridge the distances between them. When combining hopping with sliding and crowding in the simulations, we find no effect of hopping in the required parameter ranges. It should be noted that there is ambiguity in the definition of hopping in the literature, where it is sometimes defined as a non-helical displacement of the protein along the DNA while still bound to it (17,54). This would in our work correspond to a non-helical sliding mode. Such a sliding mode (defined as hopping in some other work) could have an impact on the estimated value of *D*_1_. However, we find this unlikely for two reasons: first, fully atomistic MD simulations (37) show that the repressor has a high propensity to follow the helical path. Furthermore, the agreement between the experimental value for *D*_1_ and that calculated for a helical sliding mode (51) shows that non-helical sliding does not contribute significantly, at least not *in vitro*.

### Intersegment transfer

Intersegment transfer is the process by which a protein transfers to an uncorrelated distant site as measured along the contour length of DNA via a doubly bound intermediate. This means that the transfer circumvents macroscopic dissociation where the protein would have to diffuse in 3D before finding and binding to an uncorrelated (beyond a few persistence lengths) site. The need for a doubly bound intermediate demands that the protein have two binding sites with a geometry such that a doubly bound complex is realizable. This geometrical requirement is satisfied for the LacI tetramer for which the intersegment transfer mode has been indirectly shown to occur between specific binding sites *in vitro* based on the dependence of the specific dissociation rate on the DNA concentration (57). However, to our knowledge there is no experimental evidence of intersegment transfer between non-specific binding sites neither for the LacI tetramer nor for the LacI dimer. In fact, an intersegment transfer mode has not been observed between specific binding sites for the LacI dimer either. *A priori*, based on visual inspection of the geometry, it would seem improbable that intersegment transfer would have any significant effect on the LacI dimer search kinetics. The partial unbinding of one of the two LacI dimer binding sites simply does not allow for LacI conformations which could bind to two DNA segments at the same time. Another possibility is that the LacI dimer could bind two DNA segments but that the non-specific binding strength is much stronger for the native binding site which would make intersegment transfer a very infrequent event. Although the introduction of an intersegment transfer mode does improve the search efficiency, as seen by an increased specific association rate, the results cannot be fitted well to the *in vivo* data if the intersegment transfer rate is much larger than ∼100 s^−1^. Thus, the 1D diffusion coefficient would become impermissibly high for higher intersegment transfer rates.

## CONCLUSION

We have extended the sliding model to include crowding, hopping, intersegment transfer and the possibility of traversing the specific binding site to calculate the time it takes for one LacI molecule to find its specific binding site. To account for recent *in vivo* data on the dependence between two operator sites as a function of the distance between them we also employed Monte Carlo simulations. Using the simulations and the analytical solutions as a constraint we generate solution spaces from a parameter sweep where the 1D diffusion coefficient and the degree of diffusion control were systematically varied given diverse values for the DNA occupancy, and the probability of LacI binding the specific operator site. We find that there exists a small parameter space where the model is compatible with the experimental measurements. This space is not significantly extended by allowing for hopping or intersegment transfer of the LacI dimer, which does not imply that these mechanisms do not exist, only that they do not contribute significantly in the allowed parameter space. The allowed space suggests that LacI binds the specific O_sym_-operator site with a probability larger than 0.5. We also establish that the lac repressor dimer binds non-specific DNA with a low probability at the first contact. This does however not necessarily imply an energetic barrier for binding, but rather that not all patches on the repressor or DNA will bind at contact and that steric effects may need to be considered. Finally, based on that the DNA occupancy by other proteins in the operator region seems to be ∼15%, while the overall occupancy may be higher, we propose that chromosomal DNA may have two levels of coverage by Nucleoid Associated Proteins, one fraction that are randomly associated with DNA and thus are found in the operator region where they influence binding and sliding by the repressor and one fraction of more specifically binding proteins that do not reside in the operator region and only contribute to the search time by hiding part of the chromosome from searching.

## SUPPLEMENTARY DATA

Supplementary Data are available at NAR Online.

SUPPLEMENTARY DATA
