# Supplementary material for: What matters for lac repressor search in vivo—sliding, hopping, intersegment transfer, crowding on DNA or recognition?
Source: Nucleic Acids Res. 2015 Mar 16;43(7):3454–64. doi: 10.1093/nar/gkv207 (PMC4402528; doi:10.1093/nar/gkv207)
Supplement: SUPPLEMENTARY DATA [file supp_43_7_3454__index.html]

What matters for lac repressor search in vivo—sliding, hopping, intersegment transfer, crowding on DNA or recognition? — What matters for lac repressor search in vivo—sliding, hopping, intersegment transfer, crowding on DNA or recognition? — SUPPLEMENTARY DATA 

# What matters for lac repressor search *in vivo*—sliding, hopping, intersegment transfer, crowding on DNA or recognition?

## SUPPLEMENTARY DATA

**Files in this Data Supplement:**

- SUPPLEMENTARY DATA
