# Supplementary material for: What matters for lac repressor search in vivo—sliding, hopping, intersegment transfer, crowding on DNA or recognition?
Source: Nucleic Acids Res. 2015 Mar 16;43(7):3454–64. doi: 10.1093/nar/gkv207 (PMC4402528; doi:10.1093/nar/gkv207)
Supplement: SUPPLEMENTARY DATA [file supp_gkv207_nar-00207-m-2015-File008.pdf]

What matters for lac repressor search in vivo - Sliding, Hopping, Intersegment Transfer, Crowding on DNA or Recognition?

Anel Mahmutovic, Otto G Berg and Johan Elf

Supplementary material

The sliding model describes the search of a gene-regulatory protein for its specific binding site on DNA as a two-step process where diffusion in 3D space is interspersed with 1D-diffusion along the DNA. With the detailed in vivo results now available, it is possible to identify the molecular parameters of the model. In the simulations we have accounted for sliding, hopping, intersegment transfer, crowding on DNA, and the possibility of sliding by the target without binding. Below, we will discuss the analytical description of the process, both in its original formulation and with the extensions considered in the simulations.

Nonspecific binding kinetics and hopping

a. Regular DNA distribution

In the simplest model, the DNA is considered as a smooth cylinder and the protein is a fully reactive sphere. The reaction radius ρ is the sum of the radii of the cylinder and the sphere. Nonspecific protein-DNA binding is described by the reaction radius ρ, a relative diffusion rate D_3, and the extent of diffusion control, α. All steric effects in the binding are assumed to be incorporated in the parameters ρ or α. Beyond the distance R_c from the DNA axis, the protein is assumed to have lost its correlations with a particular DNA segment and will be equally likely to bind anywhere on the DNA. If DNA has the total length $2L$ and is confined to a volume V_c, R_c can be determined from

$$2L\pi R_c^2 = V_c \qquad (1)$$

This corresponds to a regular distribution where competing DNA segments are considered as lined up in parallel at a mutual distance of ca. $2R_c$; or as a heap of cooked spaghetti filling a volume V_c where the DNA runs along at the center of each spaghetti strand of radius R_c.

A nonspecifically bound protein will dissociate with the (microscopic) rate constant λ, which leaves the protein in solution at the reaction radius $r = \rho$. Consider a protein that has just dissociated from the DNA and is present at the reaction radius, where will it rebind for the first time? The motion of the protein can be described by diffusion in cylindrical coordinates, with a radiation boundary condition at the reaction radius describing a rebinding event

$$2\pi D_3 \rho \left.\frac{\partial c}{\partial r}\right|_{r=\rho} = \kappa c(\rho) \tag{2}$$

κ is a parameter that describes the reactivity (propensity for binding) at contact. Furthermore we use an adsorbing condition at $r = R_c$ and a reflecting one at $z = \pm L$.

For a protein that has just dissociated from position $z = 0$, the return distribution density at position $z \in (-L, L)$ can be expressed as (1)

$$F(z) = \frac{1}{2L}\phi_0 + \frac{1}{L}\sum_{n=1}^{\infty}\phi_n \cos\left(\frac{n\pi z}{L}\right) \tag{3}$$

The Fourier coefficients are given by

$$\phi_n = \frac{1}{1 + \dfrac{n\beta\left[K_1(n\beta)I_0(n\vartheta) + I_1(n\beta)K_0(n\vartheta)\right]}{\alpha\left[K_0(n\beta)I_0(n\vartheta) - I_0(n\beta)K_0(n\vartheta)\right]}} \tag{4}$$

where I and K are the modified Bessel functions and $\beta = \pi\rho/L$, $\vartheta = \pi R_c/L$. The parameter α serves as a measure for the extent of diffusion control:

$$\alpha = \frac{\kappa}{2\pi D_3} = \frac{k}{2\pi D_3 \ell} \tag{5}$$

Here $k = \kappa\ell$ has been introduced such that k is a proper bimolecular association rate constant per base pair; it is the rate constant one would see if the protein were diffusing infinitely fast. For $\alpha >> 1$, the nonspecific association is totally limited by the diffusion rate. The total return probability to the same DNA segment is given by (Eq. (4) with $n = 0$)

$$\phi_0 = \frac{\alpha \ln\left(R_c/\rho\right)}{1 + \alpha \ln\left(R_c/\rho\right)} \tag{6}$$

The fraction 1-ϕ_0 of microscopic dissociations go beyond $r > R_c$ and will eventually rebind at a random position on the DNA; this is a macroscopic dissociation, sometimes referred to as an intersegmental jump Thus the macroscopic non-specific dissociation rate can be defined as

$$k_d = \lambda\left(1-\phi_0\right) = \frac{\lambda}{1+\alpha\ln\left(R_c/\rho\right)} \tag{7}$$

To give the same equilibrium constant as the microscopic rates, the macroscopic association rate constant, k_a, must satisfy $k_a/k_d = k/\lambda$. Thus,

$$k_a = k\left(1-\phi_0\right) = \frac{k}{1+\alpha\ln\left(R_c/\rho\right)} = \frac{2\pi D_3\ell}{1/\alpha+\ln\left(R_c/\rho\right)} \tag{8}$$

This is the average association rate constant for a protein starting anywhere in the solution.

During the time that the protein remains in solution, it can also diffuse in the z-direction along the DNA axis. The distribution of returns to the same segment is determined by the relation $F(z)$ as given in Eq. (3) above. During the time $1/k_d$ that the protein remains non-specifically bound macroscopically, it will make λ/k_d -1 = $\phi_0/(1-\phi_0)$ returning micro-dissociations. During these, it can diffuse and rebind at a position z some distance away, as given by the probability density $F(z)$. This process, which is an integral part of the diffusion geometry, has been called hopping (2). The return probability to the same base pair that the protein just left is given by

$$g_0 = \int_{-\ell/2}^{\ell/2} \frac{F(z)}{\phi_0}dz = \frac{\ell}{2L} + \frac{2}{\pi}\sum_{n=1}^{\infty}\frac{\phi_n}{n\phi_0}\sin\left(\frac{n\pi\ell}{2L}\right) \tag{9}$$

The probability of rebinding to a base pair that is j steps away in either direction is

$$g_j = \int_{(j-1/2)\ell}^{(j+1/2)\ell} 2\frac{F(z)}{\phi_0}dz = \frac{\ell}{L} + \frac{4}{\pi}\sum_{n=1}^{\infty}\frac{\phi_n}{n\phi_0}\sin\left(\frac{n\pi\ell}{2L}\right)\cos\left(\frac{jn\pi\ell}{L}\right) \tag{10}$$

These probabilities are conditional on the protein rebinding to the same segment that it just dissociated from, hence the normalization by ϕ_0. For α >>1, almost all rebinding events occur at the same site that the protein just left ($g_0 \approx 1$). With decreasing extent of diffusion control, α << 1, the probabilities of return become nearly independent of the distance ($g_0 \approx g_1 \approx g_2 \approx$

…). In this limit, almost all dissociations are macroscopic and the return binding position effectively randomized

b. Homogeneous distribution of nonspecific DNA

Here we will also consider an alternative – and perhaps more realistic – description of the effects from the distribution of coiled DNA. Consider the diffusion of a protein in a homogeneous distribution of non-specific DNA segments. Relative to a certain segment the diffusion can be described in cylindrical coordinates with r as the distance between the DNA axis and the protein center. The presence of other (competing) randomly distributed DNA segments can be described by a constant loss term in the diffusion equation.

$$\frac{\partial c}{\partial t} = \frac{D_3}{r}\frac{\partial}{\partial r}\left(r\frac{\partial c}{\partial r}\right) - k_a C_{ns} c \qquad (11)$$

$k_a C_{ns}$ is the product of the macroscopic nonspecific association rate constant, k_a, and the average density C_{ns} of nonspecific DNA segments. This density can be defined as above from the average concentration of DNA base pairs per unit volume as

$$C_{ns} = 2L/\ell V_c = 1/\pi R_c^2 \ell \qquad (12)$$

R_c is the same as in Eq. (1), the difference is that here the competing DNA is not assumed to be spaced regularly at distances $2R_c$, but rather with a homogeneous probability density. If the protein has just dissociated from a non-specific site, it will start just outside the DNA chain with reaction radius ρ and the initial condition is

$$c(r,0) = \delta(r - \rho^+) / 2\pi\rho \qquad (13)$$

The boundary conditions are

$$2\pi D_3 \rho \left.\frac{\partial c}{\partial r}\right|_{r=\rho} = \kappa c(\rho) \quad \text{at the reaction radius } r = \rho \text{, and} \qquad (14)$$

$$c(r,t) \xrightarrow[r\to\infty]{} 0 \quad \text{at large distance.}$$

The Laplace-transformed solution can be expressed with the modified Bessel functions of the second kind, K_0 and K_1, as

$$\tilde{c}(r,s)=\int_0^\infty c(r,t)e^{-st}dt=\frac{K_0(\mu r)}{\kappa K_0(\mu\rho)+2\pi D_3\mu\rho K_1(\mu\rho)} \tag{15}$$

where $\mu=\sqrt{\left(s+k_aC_{ns}\right)/D_3}$ and s is the Laplace variable. The return binding flux to the same DNA segment that the protein just left is determined by

$$\tilde{\varphi}(s)=\kappa\tilde{c}(\rho,s)=\frac{\alpha K_0(\mu\rho)}{\alpha K_0(\mu\rho)+\mu\rho K_1(\mu\rho)} \tag{16}$$

where $\alpha = \kappa/2\pi D_3 = k/2\pi D_3\ell$ as in Eq. (5). The total fraction that returns to the same segment is given by $\varphi_0=\tilde{\varphi}(s=0)$. Thus, if the protein dissociates from a nonspecific site with the microscopic rate λ, its macroscopic dissociation rate constant will be

$$k_d=\lambda\left(1-\varphi_0\right)=\frac{\lambda\mu_0\rho K_1(\mu_0\rho)}{\alpha K_0(\mu_0\rho)+\mu_0\rho K_1(\mu_0\rho)} \tag{17}$$

where $\mu_0=\sqrt{k_aC_{ns}/D_3}$. To give the same equilibrium constant as the microscopic rates, the macroscopic association rate constant, k_a, must satisfy $k_a/k_d = k/\lambda$. Thus,

$$k_a=k\left(1-\varphi_0\right)=\frac{k\mu_0\rho K_1(\mu_0\rho)}{\alpha K_0(\mu_0\rho)+\mu_0\rho K_1(\mu_0\rho)} \tag{18}$$

As μ_0 depends on k_a, this is an implicit relation from which k_a can be solved numerically. Although k_a in this way is not given by a closed expression, numerically the result is very close to that given by Eq. (8). To see how these two descriptions of the nonspecific DNA distributions differ, we can compare the corresponding macroscopic association rate constants from Eqs. (8) and (18), respectively.

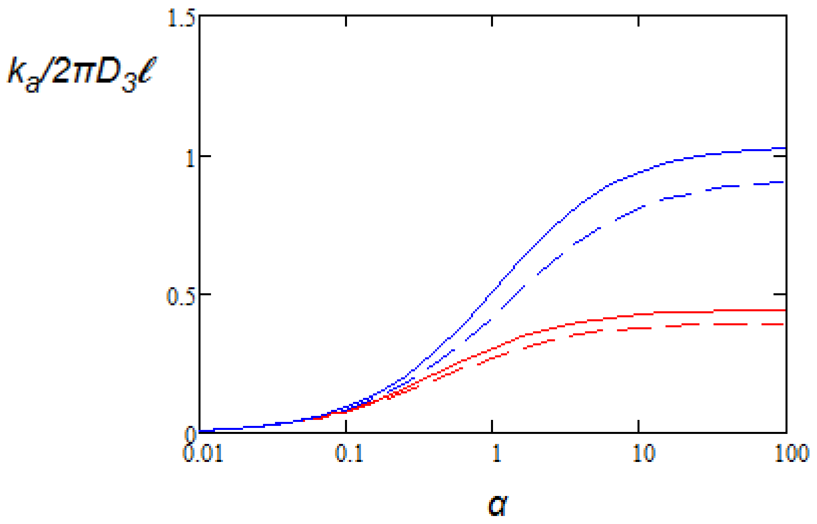

Supplementary Figure 1. $k_a/2\pi D_3\ell$ from Eq. (8) (solid) and Eq. (18) (dashed) as a function of $\alpha = k/2\pi D_3\ell$, for $2\pi D_3\ell = 6.41\times10^{-3}$ μm³/s and $R_c = 14$ nm; red curves for $\rho = 1.5$ nm and blue for $\rho = 5.5$ nm.

As a control, we can also calculate the mean time, τ_2, that the protein remains in solution after a microscopic dissociation. Integrating Eq. (15) over the unbound space in the limit $s = 0$ gives

$$\tau_2 = \int_\rho^\infty 2\pi r\tilde{c}(r,0)dr = \frac{1}{k_a C_{ns}} \frac{\mu_0\rho K_1(\mu_0\rho)}{\alpha K_0(\mu_0\rho) + \mu_0\rho K_1(\mu_0\rho)} = \frac{1-\varphi_0}{k_a C_{ns}} = \frac{1}{kC_{ns}} = \frac{R_c^2}{2\alpha D_3} \tag{19}$$

It should be noted that this is the mean time for a rebinding event anywhere on the nonspecific DNA, on the same segment or a different one. It is the inverse of the microscopic association rate constant times the density of DNA base pair. Thus, it is also independent of the form of the spatial organization of nonspecific sites as long as they are accessible; it is exactly the same result one gets from the regular distribution of DNA.

The distribution of returns to the same segment is determined by the same relation for $F(z)$ as given above, Eq. (3), but with ϕ_n replaced by

$$\varphi_n = \tilde{\varphi}(n^2\pi^2 D_3 / L^2) = \frac{\alpha K_0(\mu_n\rho)}{\alpha K_0(\mu_n\rho) + \mu_n\rho K_1(\mu_n\rho)} \tag{20}$$

where $\mu_n = \sqrt{n^2\pi^2 / L^2 + k_a C_{ns} / D_3}$. The return distribution is now determined by Eqs. (9) and (10), after replacing ϕ_n from Eq. (4) for the regular DNA distribution with φ_n from Eq. (20). As seen in Figure 1 there is very little difference between the two approaches, regular or homogeneous DNA distribution.

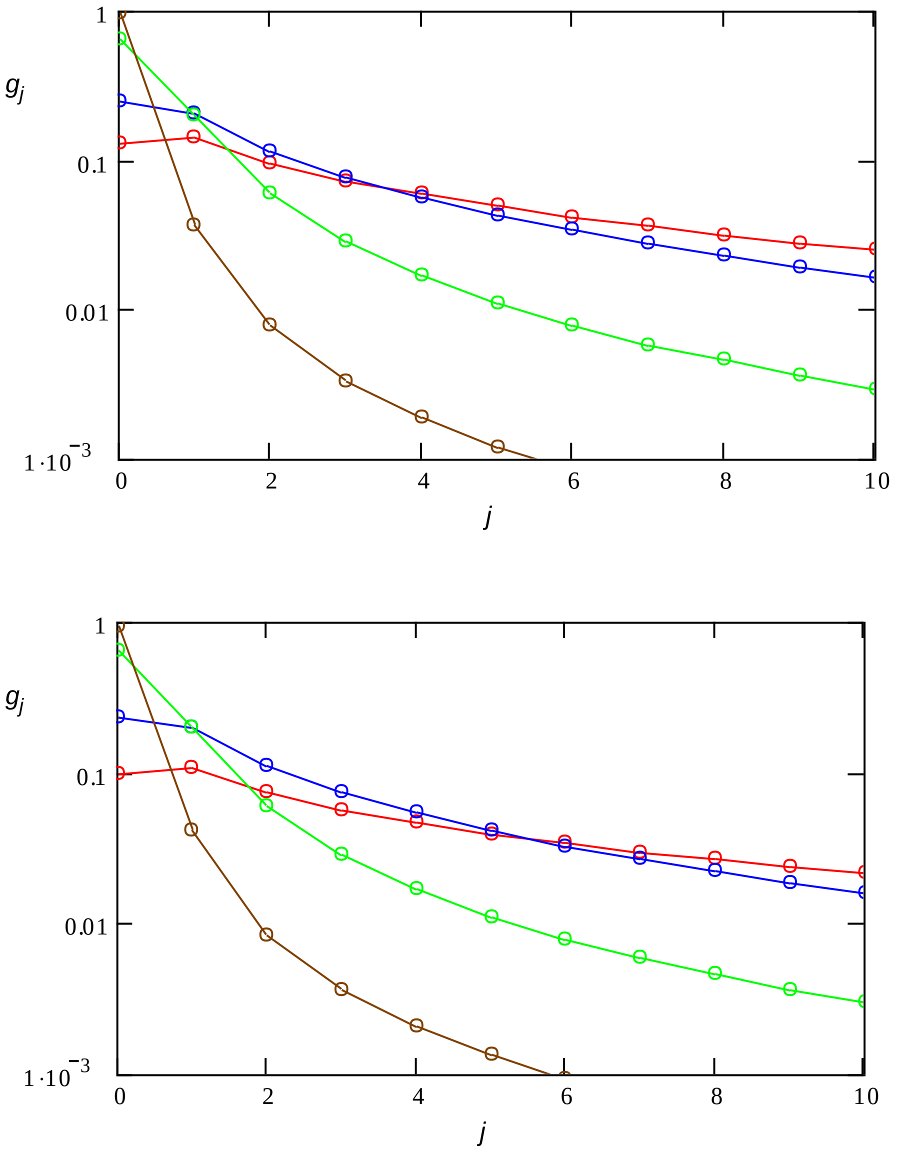

Supplementary Figure 2: Conditional return probability to a base pair *j* steps away from Eqs. (9) and (10). Top panel shows the results for a regular DNA distribution using ϕ_n from Eq. (4) and bottom panel for the homogeneous distribution as described by Eq. (20). All curves are calculated for R_c = 0.014 μm, ρ = 1.5 nm, and L = 0.34 μm; α = 0.1 (red), 1 (blue), 10 (green), 100 (brown).

Figure 3A shows the fractions of microscopic dissociations that return to the same site ($g_0\varphi_0$), and to a site 1 or 2 bp away ($g_1\varphi_0$ or $g_2\varphi_0$, respectively). At a low level of

diffusion control ($\alpha < 1$) most microscopic dissociations are macroscopic and rebind at a different DNA segment. For large $\alpha >> 1$, almost all microscopic dissociations simply return to the same site. Of the $\lambda/k_d = 1/(1-\varphi_0)$ dissociation events that occur during the macroscopic binding time ($1/k_d$), one gets away (a macroscopic dissociation) and there will be $N_H = 1/(1-\varphi_0) - 1 = \varphi_0/(1-\varphi_0)$ micro-hops that return to the same segment; of these, the fraction g_0 simply return to the same site, while the fraction $1-g_0$ execute a micro-hop to a different site. Figure 3B shows the number of micro-hops that return to the same segment (N_H, red) and to a different site on the same segment ($=N_H(1-g_0)$, blue). For small α, there are very few micro-hops ($N_H < 1$) while for large α there are many, although in this limit most of them simply return to the same site. In the diffusion-controlled limit ($\alpha >> 1$), the number of micro-hops to a different site levels off at 22 (if ρ = 5.5 nm), 18 (if ρ = 1.5 nm), and 7.8 (if ρ = 0.5 nm).

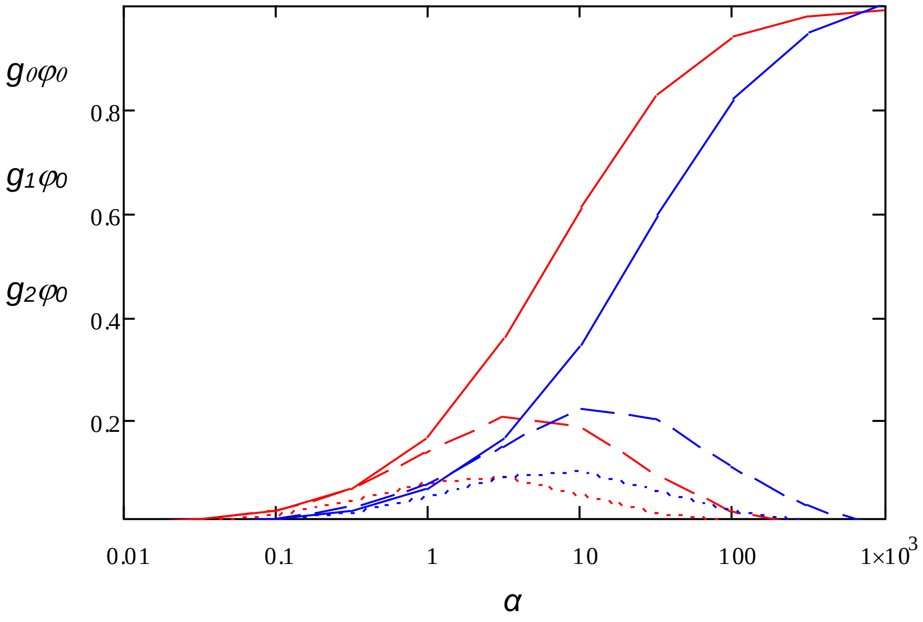

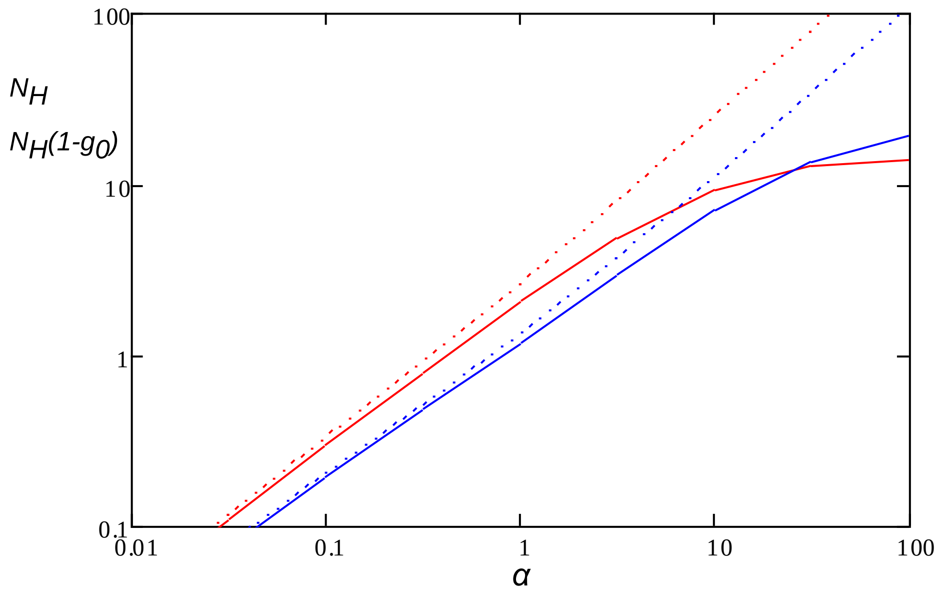

Supplementary Figure 3: Top panel (A): Return probability to the same site, $g_0\varphi_0$ (solid), to the sites one base pair away, $g_1\varphi_0$ (dashed) and two base pairs away, $g_2\varphi_0$ (dotted), as a function of the extent of diffusion control; red curves for ρ = 1.5 nm and blue for ρ = 5.5 nm. Bottom panel (B): Number of micro-hops ($N_H = \varphi_0 / (1-\varphi_0)$, dotted) and number of micro-hops to a different site (=$N_H(1\text{-}g_0)$, solid) during the macroscopic life time ($1/k_d$) of a nonspecific binding event; red curves for ρ = 1.5 nm and blue for ρ = 5.5 nm. All curves calculated for R_c = 0.014 μm, and L = 0.34 μm.

The results in Figures 1 and 2 show that the different ways of accounting for the distribution of nonspecific DNA leads to some differences in the kinetic behavior of the nonspecific binding process, particularly for α > ~1 and large values of ρ. However, the discrepancy in k_a (or k_d) as seen in Figure 1 is less than ca 20%, which shows that the explicit definition of R_c is not crucial, as long as it accounts for the average density of nonspecific DNA. Furthermore, a 20% difference in k_a translates to ca. 10% in the calculated specific association time (below), and we will stick with the simpler description given by Eqs. (4) – (8).

c. *Hopping time scale*

The mean time for a hopping event can be calculated from the return flux $\phi(t)$ for a protein that has just dissociated from the DNA cylinder. In its Laplace-transformed version this is (Eq. (5) of ref. (1)) (cf. Eq. (4) above)

$$\tilde{\phi}(s) = \frac{1}{1 + \frac{q\rho}{\alpha} \frac{K_1(q\rho)I_0(qR_c) + I_1(q\rho)K_0(qR_c)}{K_0(q\rho)I_0(qR_c) - I_0(q\rho)K_0(qR_c)}} \qquad (21)$$

Here, s is the Laplace variable and $q = \sqrt{s / D_3}$. $\tilde{\phi}(0) = \phi_0$ from Eq. (6) is the probability for return to the same segment. The mean time for such a return, conditional on one occurring, is

$$\tau_{hop} = -\lim_{s \to 0} \frac{1}{\phi_0} \frac{d\tilde{\phi}(s)}{ds} = \frac{R_c^2 - \rho^2 \left[1 + 2\ln(R_c / \rho) + 2\left(\ln(R_c / \rho)\right)^2\right]}{4D_3 \left[1 + \alpha \ln(R_c / \rho)\right] \ln(R_c / \rho)} \qquad (22)$$

After a microscopic dissociation, the TF is free in solution during the mean time τ_2 = $R_c^2/(2\alpha D_3)$, Eq. (19), before rebinding at the same or a different segment. Thus, the fraction of the free TF-molecules that are undergoing hopping at any one time is given by

$$f_{hop} = \tau_{hop} \phi_0 / \tau_2 = \frac{1 - (\rho / R_c)^2 \left[1 + 2\ln(R_c / \rho) + 2\left(\ln(R_c / \rho)\right)^2\right]}{2\left[1/\alpha + \ln(R_c / \rho)\right]^2} \qquad (23)$$

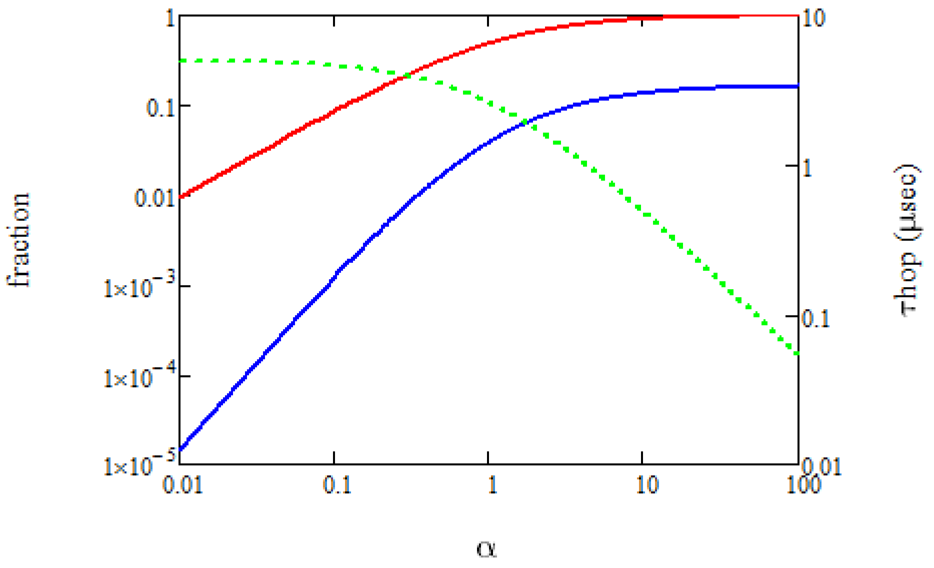

Supplementary Figure 4: Left y-scale: red line, ϕ_0, the fraction of microscopic dissociations that return to the same segment (hopping); blue line, f_{hop}, the fraction of free protein that are undergoing hopping at equilibrium. Right y-scale: green dotted line, τ_{hop}, the mean duration of a single hop. Calculations are based on R_c = 0.014 μm, ρ = 0.0055 μm, and D_3 = 3 μm^2/s.

Hopping is a possibility in all dissociation events, but in the limit of small α-values (low extent of diffusion control) it has a very low probability of occurring (Eq. (3)). In this limit, ($\alpha < 0.1$), a hopping event would last ca. 5 μsec before rebinding. In the other limit of large α, the hopping events would be more frequent, but would also be increasingly short-lived (e.g. 0.5 μsec for α = 10 or 0.05 μsec for α = 100). The fraction of the free protein that are undergoing hopping at any one time has a maximum f_{hop} = 0.16 for α >> 1 and decreases rapidly for smaller α (Figure 4).

The specific association time and the effects of hopping

Consider a transcription-regulating protein (TF) searching for one specific site embedded in a long DNA chain and present within a cell of volume V_c. While non-specifically bound, the protein can slide in a 1D diffusion process along DNA with rate D_1 (bp^2/s). In solution, the protein will be diffusing and binding/dissociating non-specifically as described above. At equilibrium, the fraction of the TF that is non-specifically bound will be

$$F_B = \frac{kC_{ns}}{\lambda + kC_{ns}} = \frac{1}{\lambda \tau_2 + 1} = \frac{C_{ns} K_{RD}}{C_{ns} K_{RD} + 1} \tag{24}$$

Here, $\tau_2 = 1/kC_{ns}$ from Eq. (19) is the mean residence time in solution after a microscopic dissociation event occurring with rate λ. This corresponds to a nonspecific binding constant (per concentration of base pairs)

$$K_{RD} = k/\lambda = k_a/k_d = \frac{F_B}{1-F_B}\frac{1}{C_{ns}} \tag{25}$$

Previously we found (1,2) that the time required to find the specific site can be expressed as

$$\tau = N_{micro}\left(\tau_2 + 1/\lambda\right) = N_{micro}/\left(\lambda F_B\right) \tag{26}$$

Note that τ_2 is a parameter that considers the mean time for all rebinding events and is independent of the spatial organization of the DNA as long as it is accessible. The mean time for the first nonspecific binding to DNA, $\tau_1 = 1/k_a C_{ns}$, is very short and has been neglected in Eq. (26); also, for the *in vivo* situation it is probably more relevant to consider the initial state as one where the protein is in a dynamic equilibrium with respect to the nonspecific DNA binding. N_{micro} is the mean number of microscopic nonspecific binding events required before the specific site is found. The result can be expressed as (Eqs. (A22)-(A24) in ref (2))

$$\tau = \frac{1}{F_B}\sum_{n=1}^{(M-1)/2} \frac{2}{4D_1 \sin^2\left(n\pi/M\right) + \lambda\left[1-\phi_n\left(M/n\pi\right)\sin\left(n\pi/M\right)\right]} \tag{27}$$

Here $M - 1 = 2L/\ell$ is the total number of nonspecific DNA sites and ϕ_n is from Eq. (4); when the nonspecific DNA is described with a homogeneous distribution, ϕ_n should be replaced by φ_n from Eq. (20). A contribution to τ that derives from the reflecting boundary condition for

the hopping events at the chain ends contributes little for long chains and has been neglected. When the hopping process is disregarded, i.e. assuming that all micro-hops return to the same site they dissociated from, the result can be calculated explicitly

$$\tau^{(-H)} = \frac{1}{F_B} \sum_{n=1}^{(M-1)/2} \frac{2}{4D_1 \sin^2\left(n\pi / M\right) + k_d} = \frac{1}{k_d F_B}\left[M \coth\left(M\Omega\right)\tanh\left(\Omega\right) - 1\right] \tag{28}$$

where $\Omega = \ln\left(1/2s + \sqrt{1+1/4s^2}\right)$ and $s = \sqrt{D_1 / k_d}$. s is the effective capture distance (bp) due to sliding; a protein that binds nonspecifically within this distance on either side of the specific site has a good chance of finding it before a macroscopic dissociation occurs. With less than 2% error if 2.5<s<<M, Eq. (28) can be approximated as

$$\tau^{(-H)} \approx \frac{1}{k_d F_B}\frac{M}{2s} \tag{29}$$

When there is neither hopping nor sliding (D_1=0, i.e. s=0), Eq. (28) is further simplified:

$$\tau^{(-H,-S)} = M / k_d F_B \tag{30}$$

This is the mean time to find a specific site if all nonspecific binding events are uncorrelated; in this case, every site must be visited on average once before the specific one is found. These results, Eqs. (29) and (30), show that sliding extends the effective target size from 1 to 2s bp. (In refs. (3) and (4), we used an approximation corresponding to 2s+1 rather than 2s in the denominator of Eq. (29) to account for direct association to the operator; this is correct when $s\to 0$ or s>>1; however, Eq. (29) is a better approximation of the full equation (28) when $s \approx 1$ or larger.)

***lac* repressor**

For the *lac* repressor in *E. coli* we use the following parameter values: $2L$ = 1.53×10^3 µm ($M = 4.5\times10^6$ bp), D_3 = 3 µm^2/s, V_c = 1 µm^3 which gives R_c = 0.014 µm, and F_B = 0.9 (or 0.7 in a few cases where marked). The mean association time measured in vivo is ca. 82 sec in a cell with an estimated number of repressors of ca. 3 to 5 per operator (3). The association time for a single repressor would then be between 250 and 400 with an average of τ = 325 sec (for four repressors per cell). We will use D_1 and the extent of diffusion control, $\alpha = k/2\pi D_3\ell$, as variable parameters; then also λ must be varied to keep F_B invariant. Figure 5 in the main text shows that hopping has little effect on the specific association time when sliding

contributes significantly or when α is small; $\tau^{(-H)}$ overestimates the association time by less than 10% if the capture distance due to sliding is $s \geq 10$.

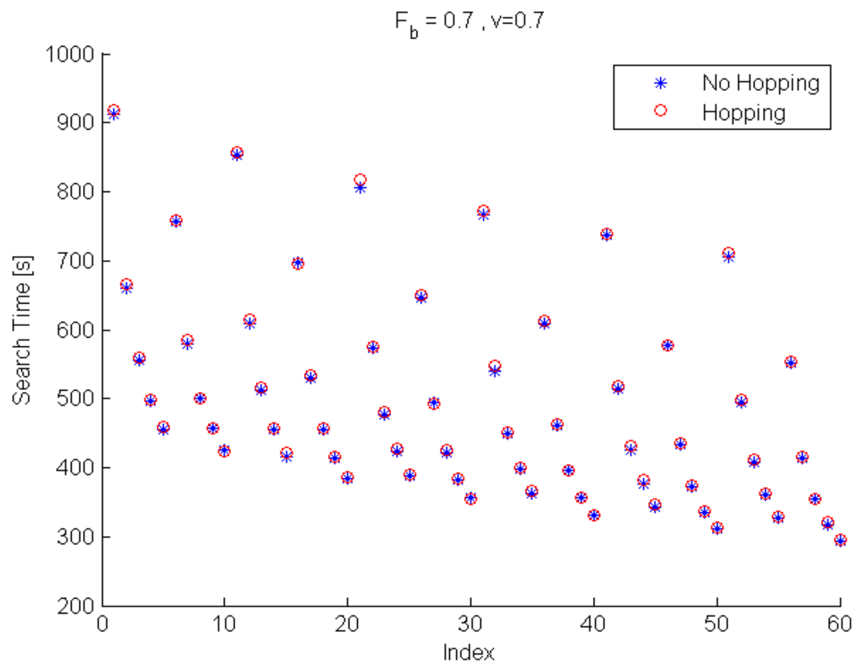

Supplementary Figure 5: The search time on the y-axis for a combination of values for the degree of diffusion control, the probability of binding the operator site and the 1D diffusion coefficient compounded into an index variable on the x-axis. The simulations were done with (red circles) and without (blue stars) hopping at a fixed value for the vacancy (70%) and for the fraction of time LacI spends non-specifically bound (70%); α was varied between [0.06,0.11] and D_1 was varied between [0.01,0.05] for p_{bind} = {0.2,1.0}. The results are overlapping – or nearly so – in all cases. The near-overlapping cases correspond to combinations of parameter values that fall outside the bounds of the acceptable solution space.

The negligible effect of hopping also holds true when including roadblocks and a recognition step as shown in Figure 5. Hopping matters mostly in the diffusion-controlled limit where α > 1. Hopping can have a large effect on τ_a only when there is no sliding and α >1. If the interaction radius ρ is as large as 5.5 nm, diffusion control and hopping could make τ ~ 100 sec even without any sliding (D_1=0, solid brown curve in Figure 5A of the main text).

To further quantify the effect of hopping on the specific search process we can calculate the effective capture distance when D_1 = 0. Thus, the effective capture length due to hopping alone would be $2s^{hop} = \tau^{(-H,-S)} / \tau^{(-S)}$ bp (Figure 6). When α <<1, $2s^{hop}$ approaches 1 and the effective target is a single base pair; as expected, hopping has no effect in this limit.

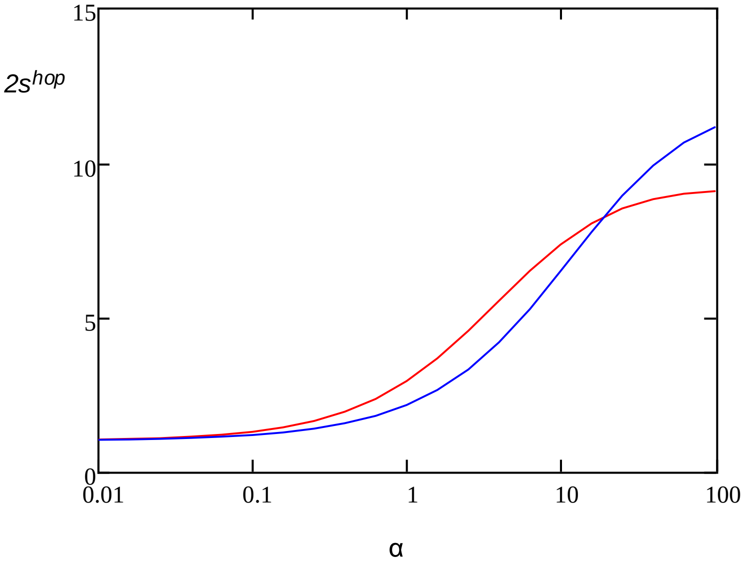

Supplementary Figure 6: The effective capture length (bp) due to hopping alone. All curves were calculated for D_3 = 3 μm^2/s, R_c = 0.014 μm, and L = 765 μm; red curve is for ρ = 1.5 nm and blue for ρ = 5.5 nm.

The occasional hops beyond the nearest-neighbor sites widen the search region. However, hopping is severely limited as it cannot extend the search region very far, as shown by the curves for $2s^{hop}$ in Figure 6. Hopping derives from the geometry of the interactions and the properties of diffusion as a volume-filling process. It is a consequence of the distinction between microscopic and macroscopic reaction rates. During a macroscopic binding event, the protein can perform microscopic dissociations, most of which quickly return to the same site while some return to a nearby site (Figures 2 and 3). In the present model, the specific target is defined as a band of radius ρ and width ℓ sitting on an inert cylinder of radius ρ. A diffusion-limited direct association would be determined by a 3D-reaction radius that corresponds to the largest linear extension of the target in 3D (5); this would be $\sim\rho$ if $\rho > \ell$. Hopping does not do any more than account for the proper diffusion limit of the specific association in the absence of facilitating mechanisms (like sliding). Thus, the maximal capture distance along the DNA due to hopping alone would be at most $\sim 2\rho$. Hopping by definition requires nonspecific dissociation; i.e. it requires that the protein does not interact with the DNA. Any motion along the DNA axis that takes place while the protein is fully or partially nonspecifically bound should be considered as a sliding motion. Conversely, any sliding step between adjacent base pairs will require a motion over a potential barrier; i.e. it will require a partial release of the protein-DNA interactions. The effect of hopping is determined primarily

by the number of micro-hops to a different site (Figure 3B), most of which go to the nearest-neighbor sites (Figure 3A). The apparent capture distance ($2s^{hop}$) due to hopping alone would be at most ca. 12 bp (Figure 6 for ρ = 5.5 nm), and much smaller if the nonspecific association is not diffusion limited or if ρ << 5.5 nm.

Thus, with ρ in the nm-range and without obstructions, it would be possible to get a reasonable association time without invoking any sliding effect. However, sliding over a distance of ca. 25 – 40 bp is required to explain the association times for tandem operators at various distances from each other. To get an effective capture length of $2s \approx 50 - 80$ bp, sliding must be the dominant process and hopping can have only a very marginal influence. In the calculations that follow, we will mostly ignore the hopping effects.

Effects of less than 100% binding probability after reaching the operator site

The results of Hammar et al. (3) suggest that the repressor will bind specifically to the operator with a probability $p_{bind} < 1$ after finding it; in other words, the repressor could simply slide over its target without actually binding. Thus, when the repressor is nonspecifically bound at the operator site it has three options: it can bind specifically with rate k_{sp}, it can slide off (left or right) with rate $2D_1$, or it can dissociate with rate k_d.

This will increase the specific association time, Eq. (28) or (29) to a single operator by the factor

$$1+\frac{\coth(\Omega)}{\gamma s^2}\approx 1+\frac{2}{\gamma s}=1+\frac{2\sqrt{k_d D_1}}{k_{sp}} \qquad (31)$$

where $s=\sqrt{D_1/k_d}$ is the sliding distance and $\gamma = k_{sp}/D_1$ is a relative measure for the recognition rate. Thus,

$$\begin{aligned}\tau_a^{(-H)} &= \frac{1}{k_d F_B}\left[M\coth\left(M\Omega\right)\tanh\left(\Omega\right)-1\right]\left(1+\frac{\coth(\Omega)}{\gamma s^2}\right)\\ &\approx \frac{1}{k_d F_B}\frac{M}{2s}\left(1+\frac{2}{\gamma s}\right)\end{aligned} \qquad (32)$$

The estimated sliding distance, s, in vivo (3) comes primarily from the ratio of the binding rate for two specific sites at distance L_0 apart relative to the binding rate for a single site. When $p_{bind} < 1$, the predicted relationship (in the absence of hopping) is

$$\begin{aligned}\frac{k_a^{(2)}(L_0)}{k_a^{(1)}} &= \frac{2\left(\gamma s^2 \tanh(\Omega)+1\right)\left\{1+\tanh\left[(L_0+1)\Omega\right]\right\}}{2\gamma s^2 \tanh(\Omega)+1+\tanh\left[(L_0+1)\Omega\right]} \\ &\approx \frac{(\gamma s+2)\left\{1+\tanh\left[(L_0+1)/2s\right]\right\}}{\gamma s+1+\tanh\left[(L_0+1)/2s\right]}\end{aligned} \tag{33}$$

Compared to the equations given in Hammar et al. (3), Eqs. (32) and (33) above have been adjusted slightly to agree with the approximation given in Eq. (29) above; this is of little consequence numerically except when $s < 1$, in which case hopping cannot be neglected anyway. For completeness we here give also the corrected association rate for a single operator that is flanked on each side by nonspecific DNA of length L_1 and L_2 bp, respectively (Eq. (4) of Hammar et al. (3)). In this case the effective capture distance will be

$$L_{eff}(L_1, L_2) = s\left[\frac{1}{\gamma s} + \frac{1}{\tanh(L_1/s)+\tanh(L_2/s)}\right]^{-1} \tag{34}$$

In the limit where both L_1 and $L_2 \to \infty$, $L_{eff} \to 2s/(1+2/\gamma s)$ as expected from Eq. (32).

Association time with crowding on the DNA

Li et al. (4) expanded the sliding model by incorporating the fact that a large fraction of the DNA in the cell is covered by protein, e.g. other transcription factors or structural proteins. Assuming that the fraction v of all nonspecific DNA is free of protein, the number of accessible DNA sites was found to be (Li et al. (4) Eq. (4))

$$M_{acc} = Mv\,\mathrm{e}^{1-1/v} \tag{35}$$

Since the parameter R_c was introduced to account for the density of accessible DNA sites, its definition will also depend on v, $M_{acc}\ell\pi R_c^2 = V_c$, or

$$R_c = \sqrt{\frac{V_c}{\pi \ell M v\,\mathrm{e}^{1-1/v}}} \tag{36}$$

The nonspecific association rate constant (per concentration of accessible DNA sites) is given by Eq. (8). Thus, the nonspecific association time is

$$\tau_a = \frac{1}{k_a C_{ns}} = \frac{1/\alpha + \ln(R_c/\rho)}{2\pi D_3 \ell} \frac{V_c}{M_{acc}} \tag{37}$$

If F_B, Eq. (24), denotes the fraction of repressors that are nonspecifically bound, the nonspecific dissociation rate is

$$k_d = \frac{1}{\tau_a} \frac{1-F_B}{F_B} = \frac{2\pi D_3 \ell}{1/\alpha + \ln(R_c/\rho)} \frac{1-F_B}{F_B} \frac{Mve^{1-1/v}}{V_c} \tag{38}$$

If the repressor does not run into any obstructions, the sliding distance (bp) would be

$$s = \sqrt{D_1 / \mathrm{k}_d} \tag{39}$$

The effective target length (bp) can be expressed as (Li et al. (4) Eq. (9))

$$L_{eff}(v) = \frac{2sv \exp(1-1/v)}{\sqrt{1 + s(1-v)\sqrt{\pi}/vd}} \tag{40}$$

Here, d is the footprint of a protein bound to the DNA, and d = 21 bp has been used in the calculations. The specific association time is found by replacing 2s by $L_{eff}(v)$ in Eq (29) (neglecting hopping)

$$\tau = \frac{Mve^{1-1/v}}{k_d F_B L_{eff}(v)} \tag{41}$$

Eqs. (38) – (41) give the mean time for a single repressor to find the target site as

$$\tau = \frac{M}{2F_B} \sqrt{\frac{1}{D_1 k_d} \left[1 + \sqrt{\pi D_1 / \mathrm{k}_d} \frac{1-v}{vd} \right]} \tag{42}$$

If there are N repressors in the cell, the time will be a factor 1/N shorter.

If the effects of crowding and of p_{bind} can be treated independently, the result in Eq. (42) could be multiplied by the factor (1+2/γs) as in Eq. (33) to give

$$\tau = \frac{M}{2F_B} \sqrt{\frac{1}{D_1 k_d} \left[1 + \sqrt{\pi D_1 / \mathrm{k}_d} \frac{1-v}{vd} \right]} \left(1 + \frac{2}{\gamma s} \right) \tag{43}$$

This holds well in the parameter regions investigated in the simulations as illustrated in Figure 7 for the case D_1=0.03$\mu m^2 s^{-1}$.

It is possible to calculate a more general expression for the joint effects of crowding and recognition by taking the average of Eq. (34) over the appropriate probabilities of finding the closest random roadblocks at distances L_1 and L_2 from the operator at each side. This gives the effective capture distance

$$L_{eff}(v,\gamma)=s\int_0^\infty dL_1\int_0^\infty dL_2\, p(L_1+d/2)\,p(L_2+d/2)\,\frac{1}{v}\left[\frac{1}{\gamma s}+\frac{1}{\tanh(L_1/s)+\tanh(L_2/s)}\right]^{-1}$$

(44)

The gap-length distribution $p(\zeta)$ is given in Eq. (3) of Li et al. (4),

$$p(\varsigma)=\frac{1-v}{d}e^{-\varsigma\frac{1-v}{dv}} \qquad (45)$$

and the effective capture distance can readily be calculated, at least numerically.

In contrast, the effects of hopping and crowding are not as easily disentangled, as also evidenced by the simulations. However, the main result remains also with crowding: when sliding is present, hopping has little influence on the specific association rate (Figure 5).

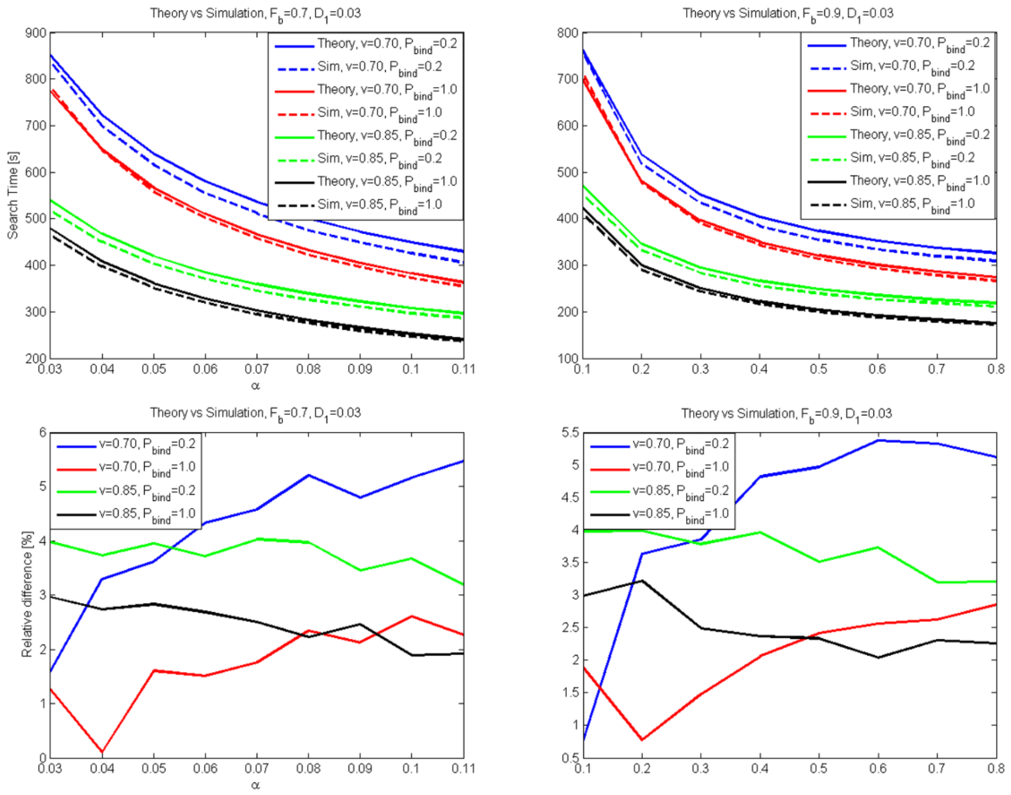

Supplementary Figure 7: Upper Row: The behavior of the extended (p_{bind} <1 and crowding) analytical formula for the association time (Eq. 40) as a function of the degree of diffusion control α for a low (left) and high (right) values for F_B in solid lines given D_1=0.03μm^{2}s^{-1}. The corresponding lattice Monte-Carlo simulations are shown as dashed lines. **Lower Row:** The relative differences between theory and simulations for the corresponding cases above.

Effect of a fixed roadblock next to the operator

Hammar et al. (3) measured the association rate to an operator where a fixed roadblock was placed right next to it. In the simple sliding model, this effectively cuts off half the access paths and would reduce the effective target length by half. However, both crowding and less than 100% recognition would influence the result. When access paths for L_1>0 are set to zero (i.e. tanh(L_1/s) ≡ 0), integration over L_2 in Eq. (44) gives the effective target length

$$L_{eff}^{(1)}(v,\gamma) = s\int_0^\infty dL_2\, p(L_2+d,v)\left[\frac{1}{\gamma s}+\frac{1}{\tanh(L_2/s)}\right]^{-1} \qquad (46)$$

The ratio of the association times with one fixed roadblock and none is given by $\tau^{(1)}/\tau = L_{eff}(v,\gamma)/L_{eff}^{(1)}(v,\gamma)$ from Eqs. (44) and (46). This agrees well with the simulations as shown in Table 2 of the main text. In the limit where a repressor binds the operator with 100% probability on arrival (i.e. $\gamma \to \infty$) one gets the simple result

$$\tau^{(1)}/\tau = L_{eff}(v,\gamma)/L_{eff}^{(1)}(v,\gamma) \xrightarrow[\gamma\to\infty]{} 2v \qquad (47)$$

Effects of intersegment transfer

Intersegment transfer is a process by which a nonspecifically bound protein can transfer to a different DNA segment without prior dissociation. This requires that the protein has a second binding site for DNA such that it can (at least transiently) bind sandwiched between two different segments. This doubly-bound complex cannot be too stable lest it would serve as a trap and slow down the search. The rate of such a transfer would be limited by the time it takes for the random segmental DNA motions to bring a second segment into a productive position and orientation to establish such a doubly bound complex. If the protein requires some conformational adjustment – e.g. partially releasing some of its binding region from its first bound segment to catch a second – the rate would be further reduced. This mechanism may well contribute significantly for the tetrameric *lac* repressor which indeed can bind two DNA segments, as has been experimentally demonstrated, at least for segments carrying specific sequences. The data discussed in this communication concern a dimeric repressor which has not shown any propensity to bind two DNA segments. Thus we expect the transfer rate – if any – to be fairly small.

In the basic model (2) it was assumed that intersegment transfer will carry a repressor from one DNA site to a random other site while it remains nonspecifically bound.

This neglects the fact that a transfer to a site nearby along the contour is unlikely because of the DNA stiffness. However, the DNA chains are very long and this neglect would be minor. With this assumption it was shown (2) that the specific association time would simply have an extra term k_{IST} (the rate of intersegment transfer) in the denominator under the summation sign in Eq. (27). As a consequence, in all equations involving the specific association time, k_d would be replaced by $k_d + k_{IST}$, and the sliding length would be $s = \sqrt{D_1 / (k_d + k_{IST})}$. Thus, with a fast intersegment transfer the effective search distance before exchange would become short, unless D_1 is very large. Although increasing k_{IST} leads to faster specific association, the concomitant decrease in the sliding length makes it difficult to fit the two-operator data without pushing D_1 up and/or k_d (i.e. α) down. Both consequences of intersegment transfer, faster specific association and the necessary increase in D_1, are illustrated in supplementary Figure 8. The semitransparent cyan region is pushed towards lower D_1 and alpha (faster association) and the minimum D_1 value with respect to the chi-squared level curves are pushed up towards higher D_1 values.

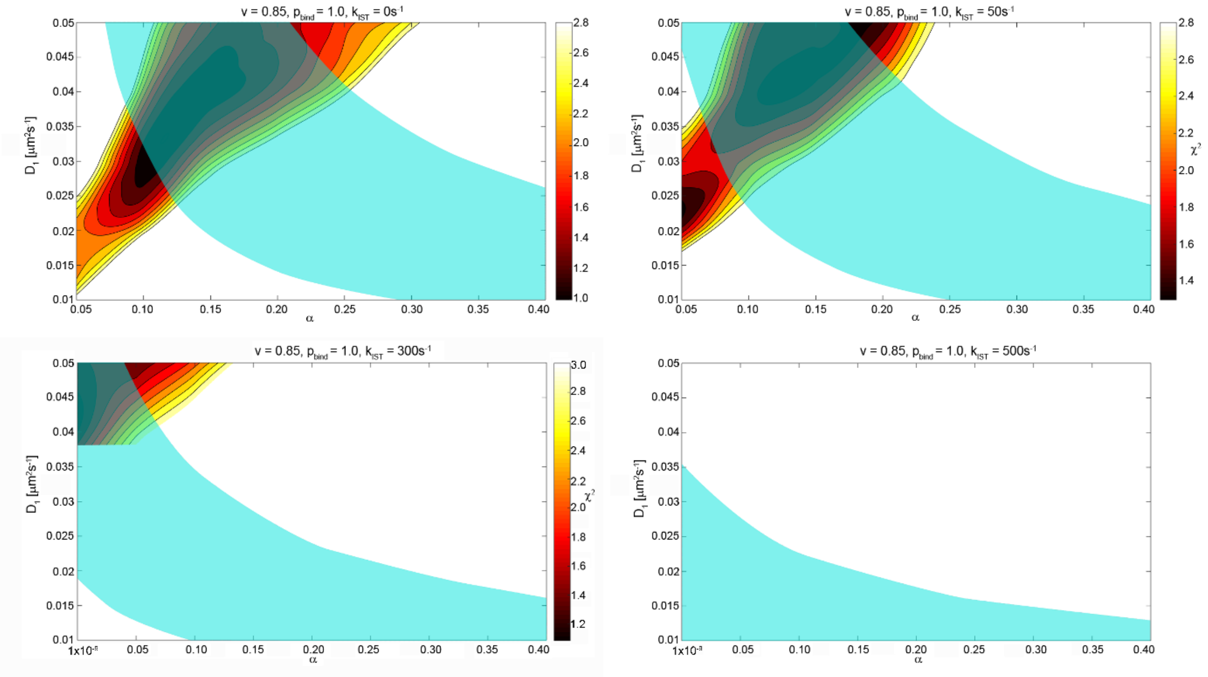

Supplementary Figure 8: The level curves correspond to chi-square values less than 3 measuring the goodness of fit of simulations to experiment for one of two operator sites at distances 25, 45, 65, 115 and 203bp. The values are calculated for combinations of D_1 and α values given the vacancy v, the probability of specific binding p_{bind} as indicated, the intersegment transfer rate k_{IST} and the fraction of non-specific binding F_B equal to 90% (Table 1). The cyan region in this figure shows where the absolute search time is in the interval 236s (3 proteins) - 416s (5 proteins).

Macroscopic and microscopic parameters

Microscopic rates refer to reaction rates for well-defined spatial arrangements of the reaction partners. The microscopic parameters of the sliding model, as developed here and previously, would be k, λ, α, D_1, ρ, and k_{sp}. These are the primary parameters but are not easily measured directly. The macroscopic parameters (k_a, k_d, τ_a, s), on the other hand, are defined from the microscopic ones and refer to average spatial configurations. In the limit where $\alpha << 1$, the distinction between the microscopic (k and λ) and macroscopic (k_a and k_d) rates disappears. The microscopic rate λ corresponds to the rate by which a protein leaves its bound state without diffusion away or reorientation. The level of diffusion control then determines the probability ($(1-\phi_0)=k_d/\lambda$) that the protein diffuses away from this newly unbound complex. The macroscopic k_a could be measured (in principle) from the mean time ($\tau_1 = 1/k_aC_{ns}$) it takes for a protein to bind to nonspecific DNA at the homogeneous concentration C_{ns} (bp per unit volume).

D_1 as measured *in vitro* could have contributions also from micro-hops along DNA. Similarly, when the capture length $s = \sqrt{D_1 / k_d}$ is used as a fitting parameter in Eq. (29) or (33), this must also include the effects, if any, of the hopping steps. There is at present no way experimentally to distinguish sliding steps from hopping steps to a neighboring base pair; both serve to expand the effective capture length.

The standard χ^2 test for the dependence between operator sites

Let $k_{exp}(x)$ be the experimentally determined rate with which LacI finds one of two operator sites at distance x={0,25,45,65,115,203} basepairs. The dependence between two binding sites is then given by $y_{\exp} = \dfrac{k_{\exp}(x)}{k_{\exp}(0)}$ where if uncorrelated $y_{\exp}$ becomes 2 otherwise $1<y_{exp}<2$.

The goodness of fit between simulations and experiments was determined using the chi-square value

$$\chi^2 = \sum_{x>0} \left(\frac{y_{\exp}(x) - y_{sim}(x)}{\sigma_{\exp}(x)} \right)^2 \qquad (48)$$

where $\sigma_{\exp}(x)$ is the experimentally determined s.e.m. value for $y_{\exp}(x)$.

References

1. Berg, OG., Blomberg, C., Association Kinetics With Coupled Diffusion – Extension To Coiled-Chain Macromolecules Applied To Lac Repressor-Operator System. *Biophys Chem*. **7**, 33-39 (1977).

2. Berg, OG., Winter, RB., von Hippel, PH., Diffusion-Driven Mechanisms Of Protein Translocation On Nucleic-Acids .1. Models And Theory. *Biochemistry*, **20**, 6929-6948 (1981).

3. Hammar, P., Leroy, P., Mahmutovic, A., Marklund, E.G., Berg, O.G., Elf, J., The lac Repressor Displays Facilitated Diffusion In Living Cells. *Science*, **336**, 1595-1598 (2012).

4. Li, GW., Berg, OG., Elf, J., Effects Of Macromolecular Crowding And DNA Looping On Gene Regulation Kinetics. *Nature Physics*, **5**, 294-297 (2009).

5. Berg, O.G., von Hippel, P.H., Diffusion-controlled Macromolecular Interactions. *Ann. Rev. Biophys. Biophys. Chem.*, 14, 131-160 (1985).
